# Optimal human respiratory simulation for exhaled gas based on CFD method

**DOI:** 10.1371/journal.pone.0313522

**Published:** 2024-11-18

**Authors:** Feng Gao, Yanfeng Li, Zhihe Su, Chunlin Wang, Haidong Wang, Junmei Li

**Affiliations:** 1 Beijing Key Laboratory of Green Built Environment and Energy Efficient Technology, Beijing University of Technology, Beijing, China; 2 School of Resources, Environment and Architectural Engineering, Chifeng University, Chifeng, Inner Mongolia, China; 3 School of Environment and Architecture, University of Shanghai for Science and Technology, Shanghai, China; Manipal Academy of Higher Education, INDIA

## Abstract

Human breathing is crucial for studying indoor environments and human health. Computational Fluid Dynamics (CFD) is a key tool for simulating human respiration. To enhance the accuracy of CFD simulations and reduce computation time, a new simulation strategy for human respiration is proposed in this paper. The effects of steady versus unsteady boundary conditions on simulation results were examined. For the unsteady boundary, sinusoidal exhalation velocities and non-inhalation gas were assumed, while the steady boundary involved constant velocities during both exhalation and inhalation phases. The jet center trajectory under different boundary conditions was analyzed and compared with experimental data. Additionally, variations in pollutant dispersion near the mouth under the two boundary conditions were discussed. Furthermore, the paper compared the calculation accuracy, calculation time and memory occupied by a single turbulence model or switching flow character models in human respiration simulation. Differences in exhaled gas vorticity and jet penetration depth across different flow models were identified. Finally, combined with the non-iterative algorithm, the optimal strategy of human respiration simulation was proposed. Results show that under the comprehensive consideration of calculation accuracy, calculation time and memory occupancy, using sinusoidal expiratory boundary conditions combined with the PISO algorithm, with the RNG *k-ε* model during expiratory phase, and switching into the laminar flow during inspiratory phase, is the optimal strategy of simulating human breathing.

## Introduction

On average, humans spend more than 70% of their time indoors every day [1], making the indoor environment a significant factor affecting human health. Research on indoor pollutants—specifically their generation, communication, and risk of exposure—have garnered considerable attention from researchers worldwide. Aerosol generated by human exhalation is one of the main indoor biological pollutants, which can cause allergies, asthma, infectious diseases and so on [2]. When there are infectious disease patients in the room, the droplets generated by human exhalation may carry a large number of pathogens and transport with the flow of indoor air, thus can exposure other indoor personnel to the risk of infection.

In terms of research on indoor respiratory droplet transmission and exposure risk under different ventilation modes, Gao and Niu [3, 4], Chao [5], Nielsen [6], etc. have published a large number of articles to investigate the effects of different ventilation methods such as displacement ventilation, mixed ventilation and individual ventilation on the spread of respiratory droplets. These studies are valuable for the design and operation of building ventilation and air conditioning systems. Among these methods, displacement ventilation offers a more stable and uniform flow field within a room. The characteristics of stable and uniform flow field are more conducive to the repetition and verification of numerical experiments. Therefore, this paper selects the displacement ventilation as indoor flow field for research and analysis.

In recent years, the simulation of human respiratory pollutants has become more accurate. Many Researchers use large eddy simulation (LES) methods to analyze particle transport of expiratory pollutants, and some new discoveries have been made. Feng [7] indicated that human body position affects human heat plume, and if the human heat plume gets thicker or faster, the aerosol generated by respiration is more difficult to penetrate. Khosronejad [8] simulated expiratory particle transport and vortex dynamics during breathing with and without face masks, and the evaporation of the particles were considered in the simulation. Ge [9] studied the effect of constant or variable area of the mouth on the penetration depth of cough jets and droplets during the whole cough process. During simulation, the droplet breakup, evaporation, dispersion and drag forces were considered in the model. Liu [10] simulated over 61000 potentially virus-laden droplets generated by coughing/sneezing. The hypotheses of buoyancy effect, bubble shape and droplet evaporation rate were verified and it can predict global puff properties such as size, velocity, droplet size distribution and so on. These above LES simulations all use sinusoidal exhalation, and Eulerian–Lagrangian approach was adopted to model the two-phase flows. Compared with Unsteady Reynolds Averaged Navier-Stokes (URANS) equations simulation, LES can achieve higher simulation accuracy. The only fly in the ointment is the above LES simulations either lack experiments, or the experimental sampling is difficult to verify numerically. By constant, the traditional URANS articles have detailed descriptions for experiments. The purpose of this paper is to verify the simulation strategy of human respiration simulation, and the accuracy of URANS can already meet the requirements when only tracer gases are simulated indoors without simulating particles.

URANS method in CFD is a powerful tool to simulate human respiration, which has been paid attention by many researchers. In CFD simulation, researchers are committed to improving the calculation accuracy of simulation, and reducing the calculation time or resources occupied by program. Some traditional improvement methods include changing the pressure-velocity decoupling algorithm [11, 12], using a more suitable turbulence model [13], using boundary conditions which are more physically meaningful or suitable simplified [14], build more accurate mathematical models [15, 16], et al. In recent years, AI-enhanced CFD has also attracted much attention from researchers. Raissi (2019) [17] introduced physics-informed neural networks (PINN), the data driven partial differential equation is realized by this neural network. This method integrates the physical law and is suitable for solving the problem of data scarcity. AI-enhance have many other applications in CFD: It can Optimize existing grids [18]; Obtain velocity and pressure which are difficult to measure through flow field image [19]; Improve the efficiency of evaluating particle dispersion [20]; Avoid iterative computation to improve the calculation speed of projection step [21], and so on. Due to space limitation, this paper only analyzes the optimization of boundary conditions, changing turbulence model and pressure- velocity decoupling in human respiration simulation.

In terms of full -model experiments, Bjørn [22] demonstrated manikins with simulated respiratory functions. Nielsen [23, 24], Olmedo [6], Liu [25], Xu [26, 27], etc. made experiments with modified manikins and published a series of articles on human respiratory characteristics, droplet transport characteristics and exposure risk under transient respiration. The experimental data in this paper was derived from this series of experiments, and the displacement ventilation tests were conducted by Olmedo et al [6].

This paper aims to Optimize human respiration simulation. For URANS, in this paper, the difference of the calculated results between the steady state boundary conditions and the unsteady boundary conditions for the same expiratory flow was compared. The two boundary conditions had different jet penetration depth, and the transportation of the tracer gas were different, which proved the necessity of sinusoidal expiratory boundary conditions in human respiration simulation. Then, during human respiration, the strategy of switching flow character model along with the exhalation flow character changing had been tested. Further, this paper discussed the effect of the above strategies combined with different pressure-velocity decoupling algorithms, and the calculation accuracy, calculation time and memory occupancy were compared, to find a better strategy for human respiratory simulation.

## Experimental model

### Summary of full-scale experiment

**Fig 1:**
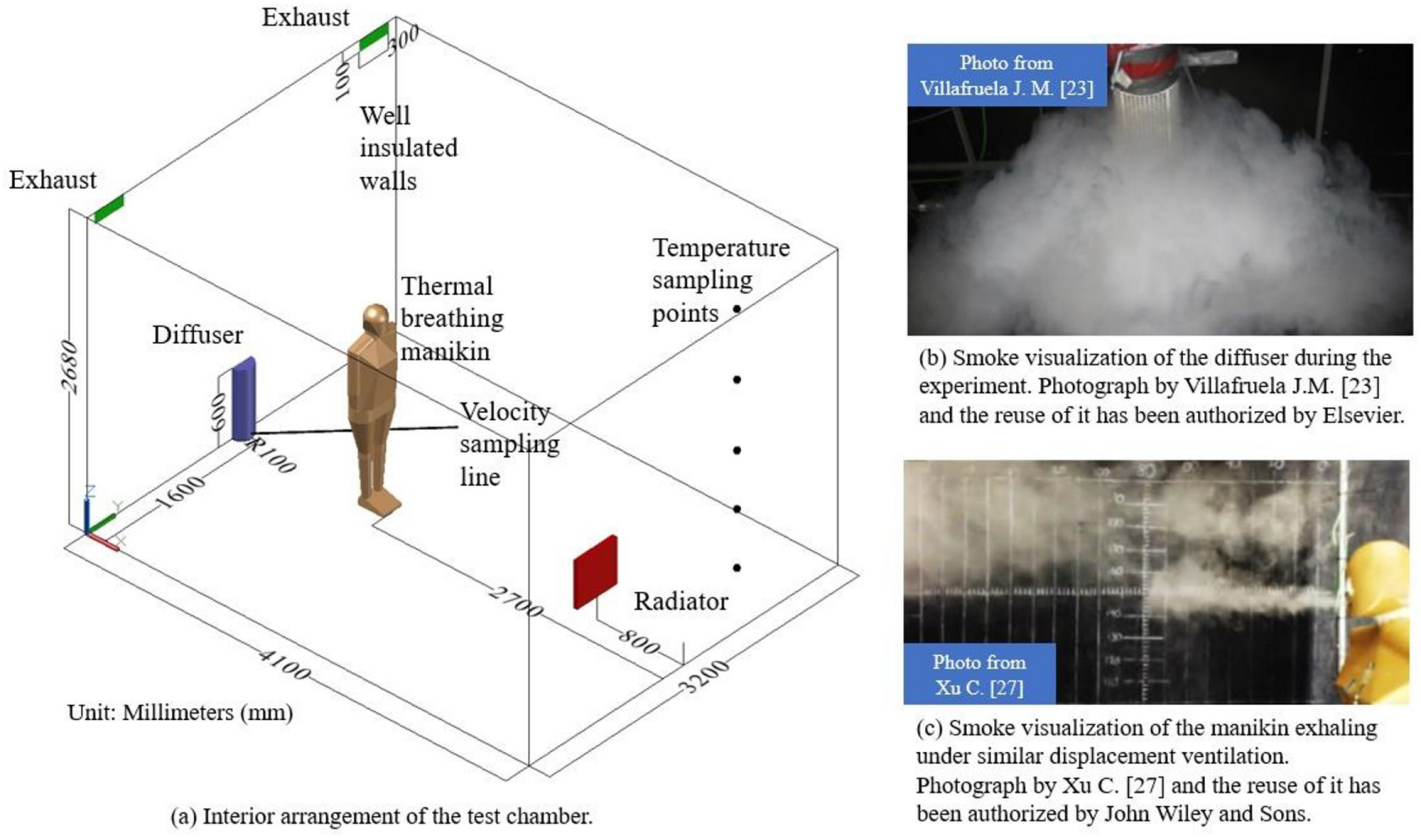
Experimental facilities. (a) Interior arrangement of the test chamber. (b) Smoke visualization of the diffuser during the experiment. (c) Smoke visualization of the manikin exhaling under similar displacement ventilation.

The contents and experimental data from previous studies [6, 23] were used in this study. This experiment recorded a 4.1 m long, 3.19 m wide, and 2.68 m high room. Displacement ventilation was performed with a semicircular diffuser and two exhausts, their location are shown in Fig 1(a). A 1.68-meter-high single manikin with real breathing stood on the symmetric plane of the Y axis of the room, and the velocity and pressure values of the sampling points were obtained during manikin respiration. This experiment also used the N_2_O as a tracer gas to analyze the penetration depth of the exhalation jet, moreover, the exposure risk of exhaled gas pollutants can be reflected by the N_2_O mass fraction.

In Fig 1, the radius of the semi-circular diffuser was 100 mm and the height was 600 mm. Both the two exhausts were 300 mm long and 100 mm high. The room and interior layout were symmetrical. The air velocity of the diffuser was 0.2892 m/s, and the temperature of supply air was 16.1°C. The air in test chamber was discharged through two exhausts, and the number of air changes in test chamber was 5.6 times per hour. The radiator was on the symmetry plane with a heat power of 394 W (679.32W/m^2^). The heat power of manikin was 94W (64.60W/m^2^). The airflow parameter of breathing function is shown in Table 1. Before the manikin breathing test, the indoor thermal environment reached a steady state, and the average room temperature was 21±1°C. The average temperature of the room remained stable during the experiment.

**Table 1:** Boundary conditions used during the simulations.

Object	Boundary type	Velocity [m/s]	N_2_O mass fraction	Heat [W/m2]	Temperature [°C]
**Diffuser**	Velocity inlet	0.2892	—	—	16.1
**Exhaust**	Pressure outlet	—	—	—	—
**Walls**	Wall	—	—	Adiabatic	—
**Radiator**	Wall	—	—	679.32	—
**Manikin body**	Wall	—	—	64.60	—
**Manikin mouth (transient)**	Velocity inlet	If v>0,	0.0270	—	33.8
		v = 4.5*sin (1.79*t).			
		If v<0, v = 0.			
**Manikin mouth (steady)**	Velocity inlet	2.25	0.0172	—	29.2

The reuse of experimental boundary conditions has been authorized by Elsevier.

Under displacement ventilation conditions, the flow field around the semicircular diffuser was more uniform than other ventilation modes, and it was very beneficial for both experiments and simulations. Smoke visualization of the diffuser during the experiment is shown in Fig 1(b). Under similar experimental conditions, the smoke visualization of the manikin breathing is shown in Fig 1(c).

### Boundary conditions and meshing

The simulated boundary conditions were set strictly in accordance with literature [23], as shown in Table 1.

**Fig 2:**
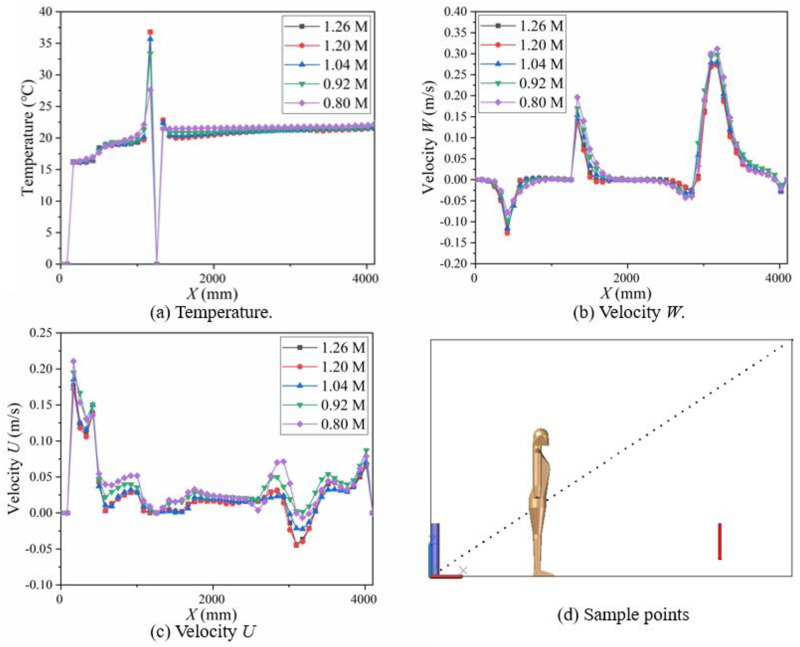
Grid independence test for: (a) Temperature, (b) Velocity W, (c) Velocity U, and (d) Sampling points in the X-Z plane.

In order to get mesh-independent solutions, under the steady boundary conditions in Table 1, we tested the calculation results of 0.8 million grids, 0.92 million grids, 1.04 million grids, 1.2 million grids and 1.26 million grids. A total of 100 sampling points were taken from the line between point (0, 1600, 0) and point (4100, 1600, 2680), The comparison of different meshing on calculated temperature, velocity *U*, velocity *W* are shown in Fig 2. The grid independence test showed that the calculated values were very close in the calculation results of 1.2 million grids and 1.26 million grids. 1.2 million grids had passed the grid independence test and it was selected for simulation.

**Fig 3:**
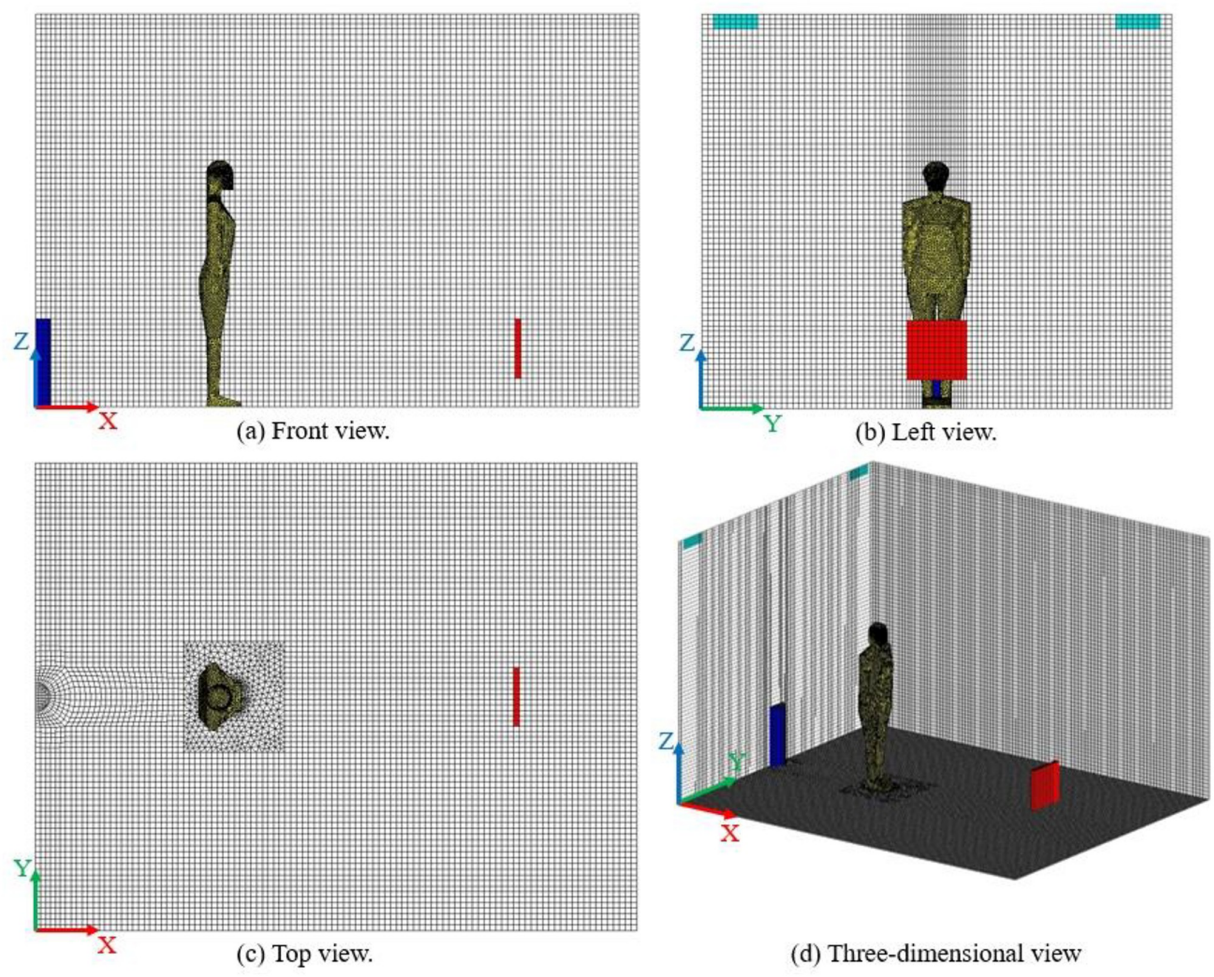
Meshing of the test chamber.

The meshing of the test chamber is shown in Fig 3.

### Meshing details

**Fig 4:**
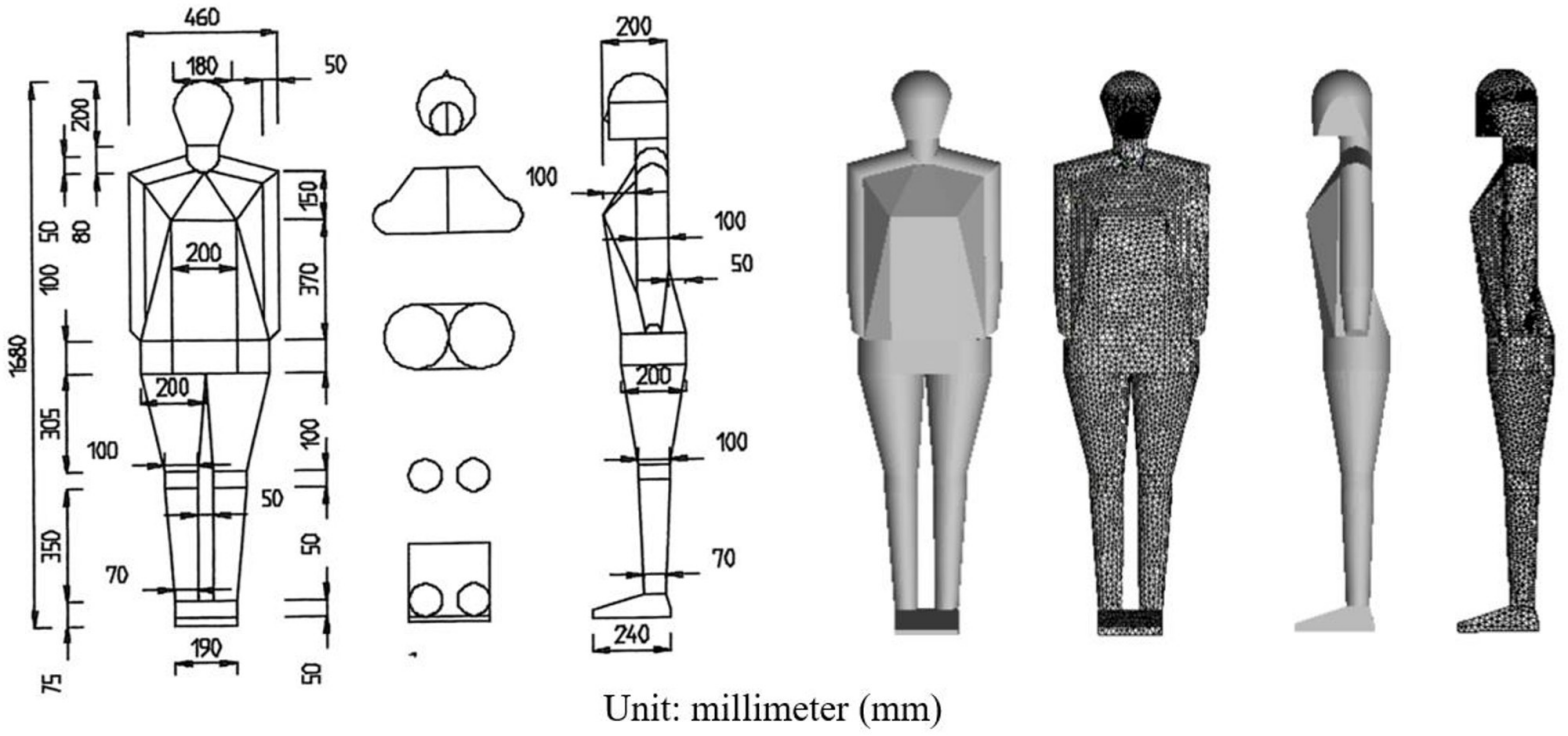
Drawing of the manikin [23] (left) and the geometry and meshing body of CSP (right). The reuse of the human model drawings has been authorized by Elsevier.

The human body produces heat plumes in an air-conditioned room. For the computer simulated persons (CSPs) in built environment, simplified geometry of CSPs can greatly reduce the amount of numerical calculation, and it is very attractive to both researchers and engineers. However, some cases are not suitable for simplified CSPs. On one hand, the difference of human body geometry only affects the prediction of airflow in the plume field, while the influence on the temperature field is very limited [28–30]. From the perspective of thermal comfort, the simplification of human body shape would not affect the evaluation of PMV and PPD [31]. On the other hand, although the simplification of CSPs only affect the prediction of airflow in the plume field, it would expand the prediction error of the pollutant transportation in the whole domain during calculation [30]. Ai and Melikov [14] pointed out that different boundary conditions would directly affect the simulation results of airborne propagation of droplet nuclei, and the shape of CSPs was one of the boundary conditions that matters. In view of the important impact of the CSPs geometry on the pollutants transport, in this paper, the CSP model was carried out strictly in accordance with the manikin drawings in the series articles [22–24]. The CSP model established according to the original literature and the meshing body are shown in Fig 4, the length unit is millimeter (mm).

In this paper, a hybrid mesh was used. For drawing human body mesh, tetrahedral mesh can use less mesh to fit surfaces than structural mesh, and often with much higher calculation accuracy, faster convergence. The tetrahedral mesh was used around the human body to capture the geometric features of the CSP, and the hexahedral mesh was used in the rest of the room. The triangular shell mesh on the CSP surface was automatically generated by the patch independent method, and the tetrahedral mesh near the CSP was automatically generated by octree method. Octree algorithm divides the space cube of the whole scene into 8 sub-cube grids to form an octree, after the scale threshold is selected, the octree continuously dissects the sub-cube grid until the conditions are met [32]. Therefore, in the tetrahedral mesh generated by the Octree algorithm, at the interface of the large tetrahedron and the small tetrahedron, the large tetrahedron side length is twice that of the small tetrahedron side length. If the side length of the global maximum grid is set to the power of 2, the tetrahedral mesh can obtain the side length with integer number, which can reduce the error of numerical calculation. In this paper, 1.2 million grids were tested through grid-independent solutions. This grid solution set the global maximum mesh size to 40 mm. After the octree algorithm was used to divide the grid for 5 times, the maximum mesh size was 1.25 mm. The mesh size was suitable for numerical calculation.

**Fig 5:**
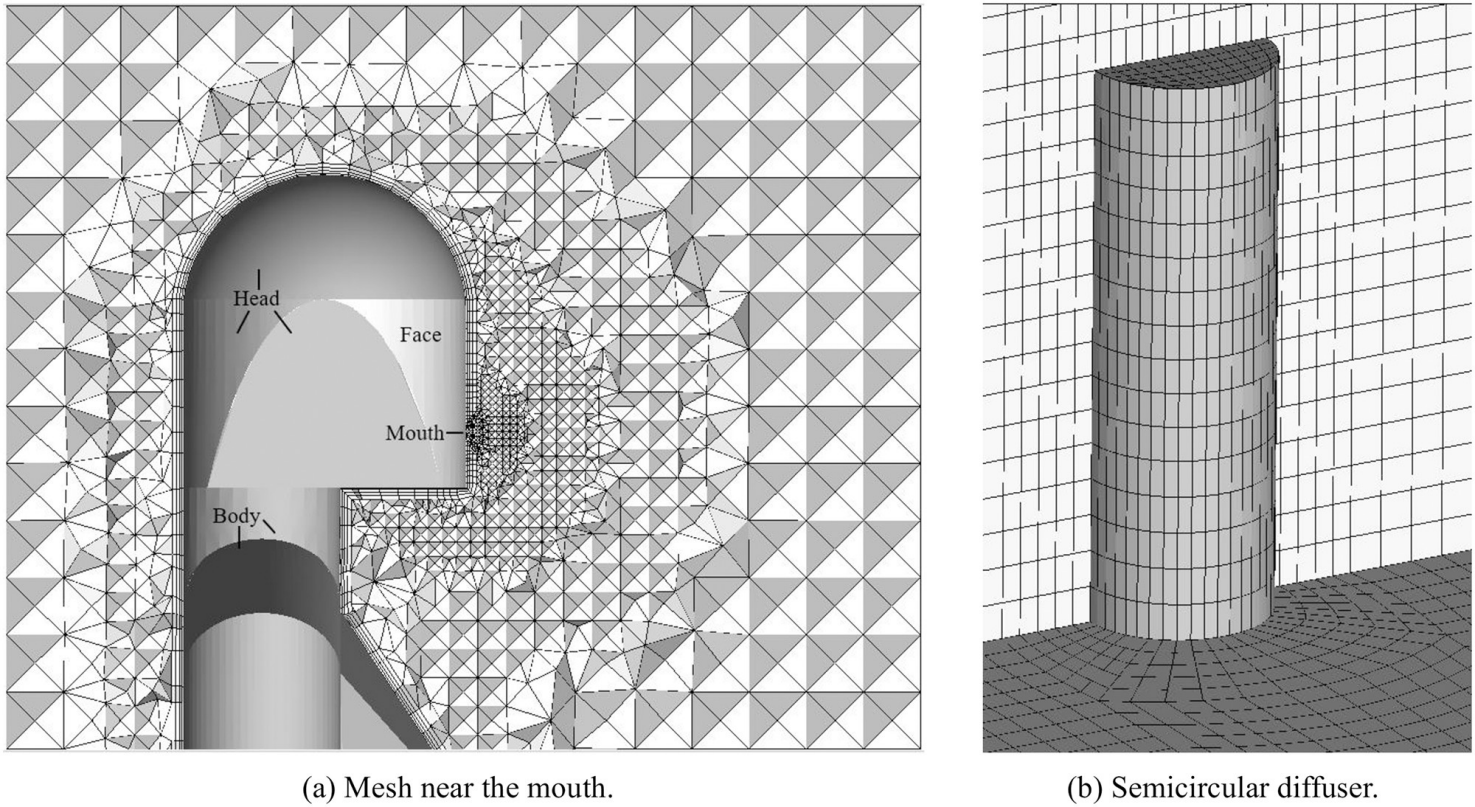
Meshing details. (a) Mesh near the mouth, (b) Semicircular diffuser.

The grid division near the human mouth is shown in Fig 5(a). The mouth was used as the velocity inlet of the calculation domain, and no boundary layer was set. Four boundary layers were set for the head, face and body of the manikin. The mesh height of the first layer was 1.2 mm, and the boundary layer height ratio was 1.3. Near the mouth, the mesh density was increased. The grid settings near the manikin are shown in Table 2. The grid division of the diffuser is shown in Fig 5(b). In this paper, the structure grid was used to describe the semicircular diffuser. Compared with tetrahedral grid, the mesh quality for semicircular diffuser was substantially improved.

**Table 2:** The grid settings near the manikin.

Object	Mesh height of the first layer [mm]	Height ratio of the boundary layer	Number of the boundary layers	Maximum size of shell mesh [mm]
**Mouth**	-	-	0	2.4
**Face**	1.2	1.3	4	10.4
**Head**	1.2	1.3	4	26
**Body**	1.2	1.3	4	40

The final number of grids was 1.2 million, including 493,000 tetrahedral mesh and 581,000 hexahedral mesh and 126,000 other mesh, with a total of 718773 nodes. The original literature [23] used 0.87 million grids to simulate this experiment, and the grids in this paper were denser than those in the original literature.

### Numerical model and algorithm selection

In this paper, a numerical model was established using mass conservation, momentum conservation, energy conservation, component conservation and turbulence equations, and the radiation was calculated with S2S model.

### Flow character model

Taking the N-S equation in the x direction as an example for analysis, and its momentum equation is shown as Eq (1).


$$\frac{\partial u}{\partial t}+\frac{\partial uu}{\partial x}+\frac{\partial uv}{\partial y}+\frac{\partial uw}{\partial z}=-\frac{1}{\rho }\frac{\partial p}{\partial x}+\nu \nabla^{2}u+f$$ (1)

The velocity vector of a particle in the flow field can be decomposed into the sum of the time -mean value and pulsation value of the velocity in each direction. So, it is defined that *u* = *U* + *u*′, in which u represents the instantaneous velocity in the x direction, U represents the average time velocity in the x direction, and u’ represents the pulsation velocity in the x direction. So as the same definition for *v* = *V* + *v*′, *w* = *W* + *w*′. With this method, the velocity in each item of Eq (1) can be re-derived and that is Reynolds time-average method. The derivative of each item containing velocity in Eq (1) is shown in Eqs (2)–(6).


$$\bar{\frac{\partial u}{\partial t}}=\frac{\partial \bar{u}}{\partial t}=\frac{\partial U}{\partial t}$$ (2)

$$\bar{\frac{\partial uu}{\partial x}}=\frac{\partial \bar{uu}}{\partial x}=\frac{\partial \bar{\left(U+u\prime )(U+u\prime \right)}}{\partial x}=\frac{\partial UU}{\partial x}+\frac{\partial \bar{u\prime u\prime }}{\partial x}$$ (3)

$$\bar{\frac{\partial uv}{\partial y}}=\frac{\partial \bar{uv}}{\partial y}=\frac{\partial \bar{\left(U+u\prime )(V+v\prime \right)}}{\partial y}=\frac{\partial UV}{\partial y}+\frac{\partial \bar{u\prime v\prime }}{\partial y}$$ (4)

$$\bar{\frac{\partial uw}{\partial z}}=\frac{\partial \bar{uw}}{\partial z}=\frac{\partial \bar{\left(U+u\prime )(W+w\prime \right)}}{\partial z}=\frac{\partial UW}{\partial z}+\frac{\partial \bar{u\prime w\prime }}{\partial z}$$ (5)

$$\bar{\nabla^{2}u}=\nabla^{2}\bar{u}=\nabla^{2}U$$ (6)

Substituting the above items into Eq (1), the Reynolds time-average momentum equation in the x direction can be obtained, shown as Eq (7).


$$\frac{\partial U}{\partial t}+\frac{\partial UU}{\partial x}+\frac{\partial UV}{\partial y}+\frac{\partial UW}{\partial z}=-\frac{1}{\rho }\frac{\partial p}{\partial x}+\nu \nabla^{2}U-(\frac{\partial \bar{u\prime u\prime }}{\partial x}+\frac{\partial \bar{u\prime v\prime }}{\partial y}+\frac{\partial \bar{u\prime w\prime }}{\partial z})$$ (7)

In Eq (7), the force represented by u’u’, u’v’, and u’w’ is Reynolds stress [33]. In the three-dimensional Cartesian coordinate system, Reynolds stress has a total of 6 items, which constitutes a symmetric second-order tensor, shown as Eq (8).


$$-\left[\begin{array}{ccc} \bar{u\prime u\prime } & \bar{u\prime v\prime } & \bar{u\prime w\prime }\\ \bar{u\prime v\prime } & \bar{v\prime v\prime } & \bar{v\prime w\prime }\\ \bar{u\prime w\prime } & \bar{v\prime w\prime } & \bar{w\prime w\prime } \end{array}\right]$$ (8)

To address the Reynolds stress in each term, various turbulence models have been proposed, including the most famous series of *k-ε* model and the improved RNG *k-ε* model. The standard *k-ε* model is suitable only for fully developed turbulence, while the RNG *k-ε* model increases the application range of turbulence simulation by renormalization group calculation, and can be extended to the simulation of low Reynolds number flows by solving differential formula for effective viscosity. During the expiratory phase, the flow of fluid is characterized by turbulence. Numerous literatures [4, 34] have shown that RNG *k-ε* model has better computational accuracy than standard *k-ε* model in turbulence simulation, Therefore, the RNG *k-ε* model was selected to simulate the expiratory phase in this paper.

In laminar flow, the fluid movement is smooth and does not exhibit pulsating velocity. During the inspiratory phase, the flow rate of exhaled gas changes from peak to 0 L/s, and the flow characteristics of the exhaled gas changed from turbulent flow to laminar flow when there is no driving force. The RNG *k-ε* model, which is suitable for low Reynolds number flows, would decrease the robustness of the differential equations and increase the computational cost. Laminar flow might be used to simulate the exhaled gas during inspiratory phase. One objective of this paper is to test whether it is suitable to switch flow character model to laminar flow during the inspiratory phase of simulated breathing.

The enhanced wall treatment and RNG *k-ε* model were used together [35], and this method is an universally applicative method for the boundary layer near the wall. It is not necessary to set the wall function when simulating the laminar flow during inspiratory phase.

### Pressure-velocity decoupling algorithm

**Fig 6:**
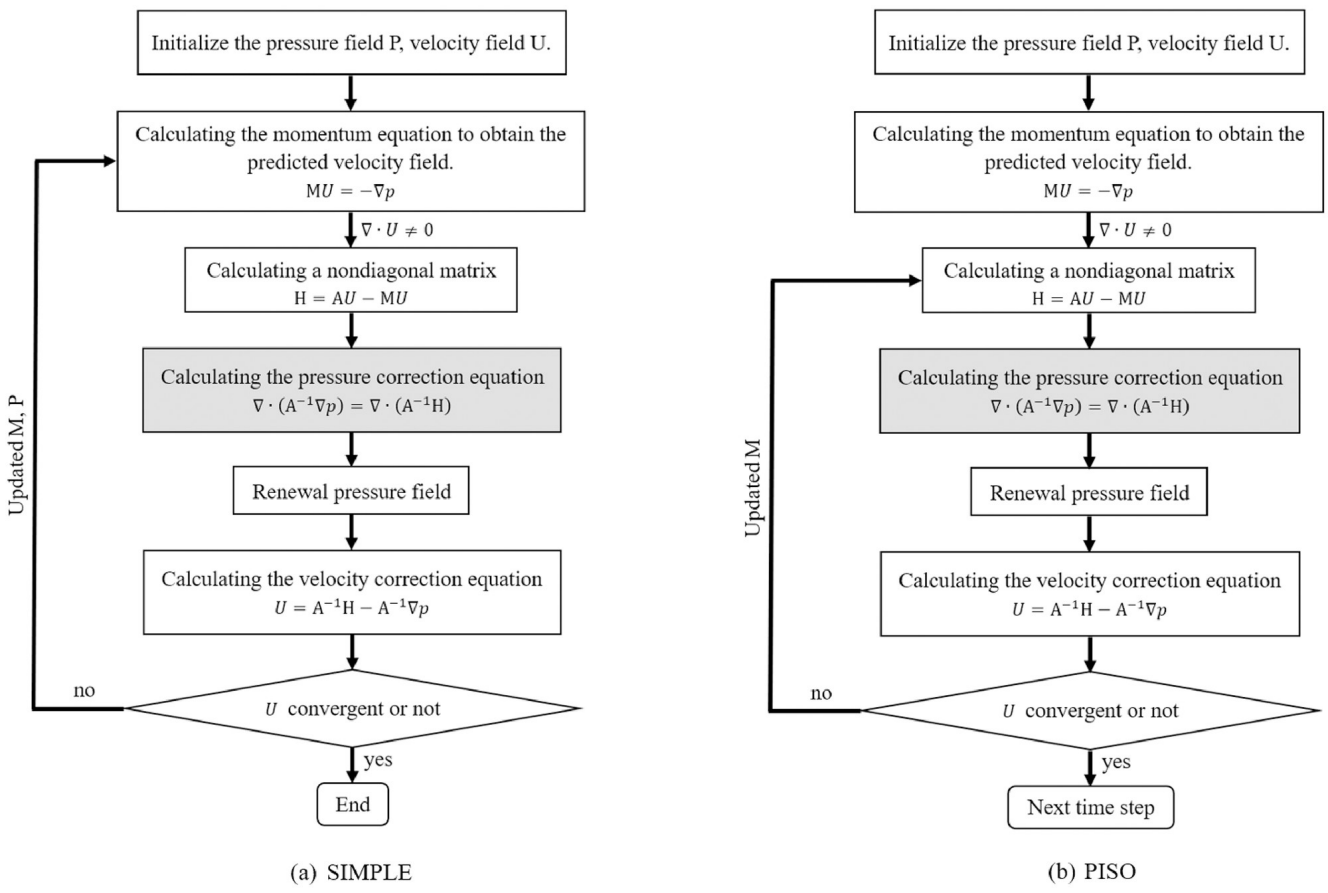
Flowchart of SIMPLE algorithm and PISO algorithm. (a) SIMPLE, (b) PISO.

In the flow field, pressure and velocity influence each other, and the decoupling algorithm of pressure and velocity directly affects the calculation accuracy and efficiency. Two primary decoupling methods are commonly used by CFD software: fully-implicit coupled solver and semi-implicit separation solver. Full implicit coupled algorithm can use a large time step, it has high convergence speed and high calculation accuracy and needs to occupy a large memory for program, many steady simulations use this algorithm to solve. However, if coupled algorithm is used in unsteady simulation, it is difficult to accept too much computation and too long calculation time. In the unsteady simulation, the separation algorithm is generally used for calculation. Separation algorithms can be divided into two categories: iterative algorithms and non-iterative algorithms. SIMPLE algorithm is an iterative algorithm, and its flowchart is shown in Fig 6(a) [36]. PISO algorithm is a non-iterative algorithm [37], and its flowchart is shown in Fig 6(b) [38, 39].

In Fig 6, both algorithms include pressure correction equations of the same format. The pressure correction equation is a Poisson equation, and its numerical solution requires a large amount of calculation time, sometimes even comes up to 80% of the entire simulation time [40]. In unsteady simulation, reducing the number of iterations, especially the number of calculations for the pressure correction equation, can significantly reduce the calculation time.

The SIMPLE algorithm achieves the convergent solution through multiple iterations. In the case of unsteady simulation, the SIMPLE method requires several iterations at each time step, classifying it as an iterative algorithm [41]. The iterative algorithm can consume substantial computing resources. In contrast, PISO algorithm is specially applied to unsteady simulation, and the algorithm usually only needs 2–3 iterations in each time step to reach the convergent solution, making it a non-iterative algorithm. In iterations of each time step, the PISO algorithm reduces the solving times of the first step of momentum equation, so the computation amount of each iteration in PISO is also smaller than that of SIMPLE algorithm. For unsteady simulation especially in small time steps, PISO algorithm has small calculation amount, high calculation accuracy and fast rate of convergence, and it has been adopted by many CFD software.

Another objective of this paper is to explore the effects of non-iterative algorithms combined with switching flow character model simulation strategies on human respiration simulation. Therefore, in this paper, coupled algorithm, SIMPLE algorithm and PISO algorithm were respectively used to simulate the unsteady human respiration. The calculation time, calculation accuracy and occupied memory of different methods were compared to find the optimal human respiration simulation strategy.

## Results and discussion

### Different results between steady or unsteady boundary conditions

The integral of expiratory flow under the sinusoidal unsteady boundary condition was equivalent to that under the steady boundary condition. The unsteady expiratory boundary condition simulated the mouth as sinusoidal velocity inlet, that was 4.5·sin (1.79·t) m/s, the mass fraction of N_2_O was 0.0270, and the expiratory temperature was 33.8°C. While in the steady case, the flow of oral exhalation was 2.25 m/s, the mass fraction of N_2_O was 0.0172, and the expiratory temperature was 29.2°C. Inspiratory flow was not considered for either boundary condition. If the time turned from expiration to inspiration, no gas flowed from the mouth, and the velocity was set for 0 m/s.

In the steady case with constant boundary condition, the convergence solution was relatively difficult to be achieved under the SIMPLE algorithm, which was consistent with reference [23]. However, if the coupled algorithm was used, a convergent solution could be achieved. In this example, the coupled solver was more suitable for calculating steady state than the separate solver. For the calculation results, the average room temperature under the steady boundary condition with coupled algorithm was 20.20°C, while the result in the original document [23] which under unsteady boundary condition was 21.80°C. The average room temperature measured in the experiment should be 21±1°C. The deviation rate of the mean temperature in the original literature was 3.81%, and the deviation rate of mean temperature in this paper was same [23].

For unsteady simulation, the steady simulation results were taken as the initial conditions, and the transient velocity inlet in Table 1 were used for unsteady simulation. The unsteady simulation performed 6 breathing cycles, corresponding to a true duration of 21.06 s. After 5 breathing cycles (17.55 s), the kinetic energy of the steady -results -exhaled -gas which under the initially set had already been dissipated, and the influence of the initial value was almost negligible. Therefore, the time-average value of the flow field of the 6th breathing cycle was selected for analysis in this paper. The coupled algorithm was used in this section. The time step was 0.01 s and the maximum number of iterations per time step was 25. The calculated results converged and satisfied the mass conservation.

**Fig 7:**
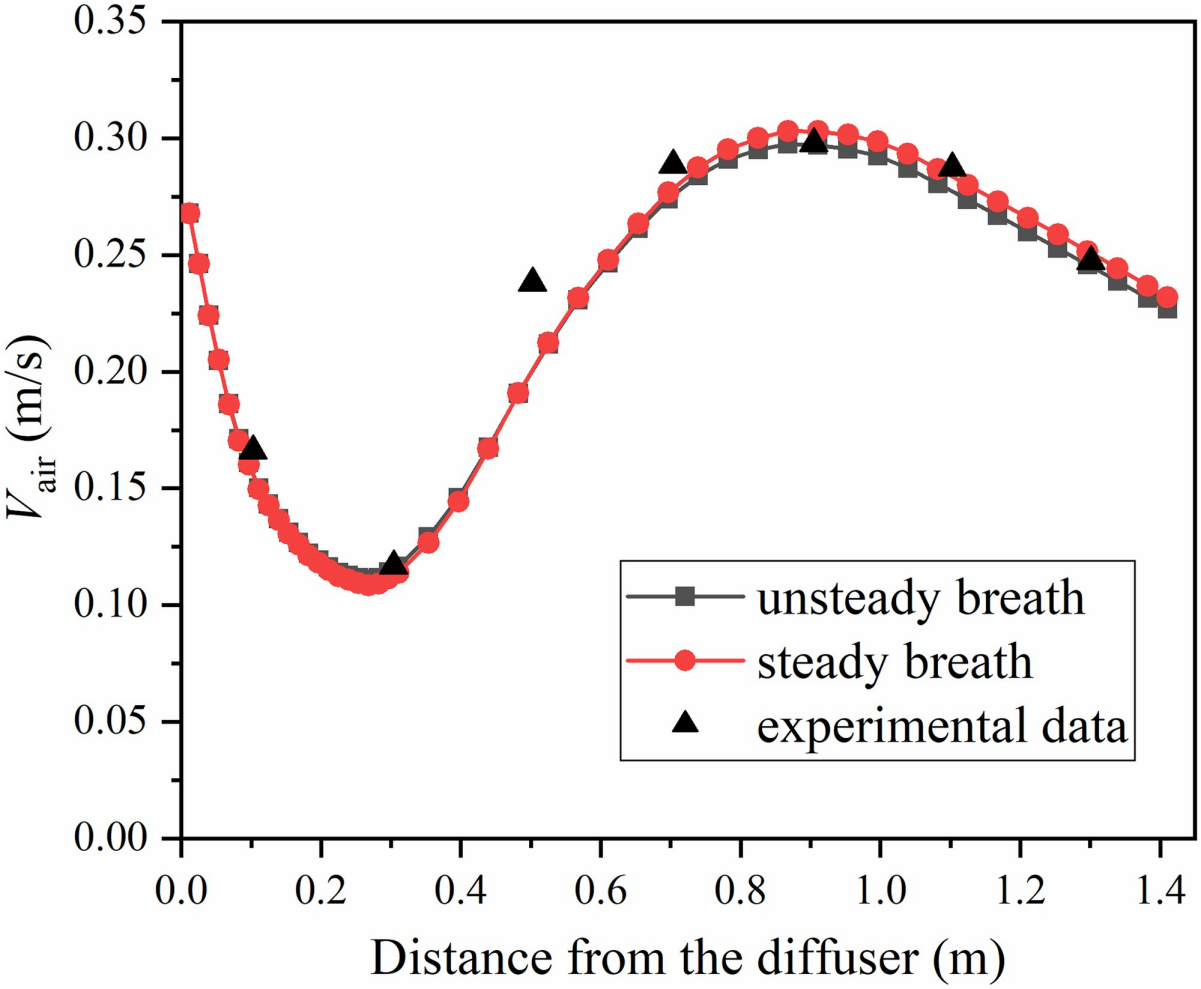
Simulated velocity values of the two boundary conditions and the experimental data. The experimental data came from reference [23], and the reuse of data has been authorized by Elsevier.

The comparison of the simulated velocity values among the two boundary conditions and the experimental data is shown in Fig 7. The sampling points were located on a line between the coordinates (0.1, 1.595, 0.04) and (1.1, 2.595, 0.04), and the calculated value approximated the projection point velocity value at 0.01m height. In Fig 7, the horizontal axis represents the distance from the sampling point to the diffuser, and the vertical axis shows the average velocity at the sampling point. Both boundary conditions could predict the trend of velocity variation, and the error increased a little in the region affected by body thermal plume. Both the two boundary conditions were within the acceptable range of accuracy.

**Fig 8:**
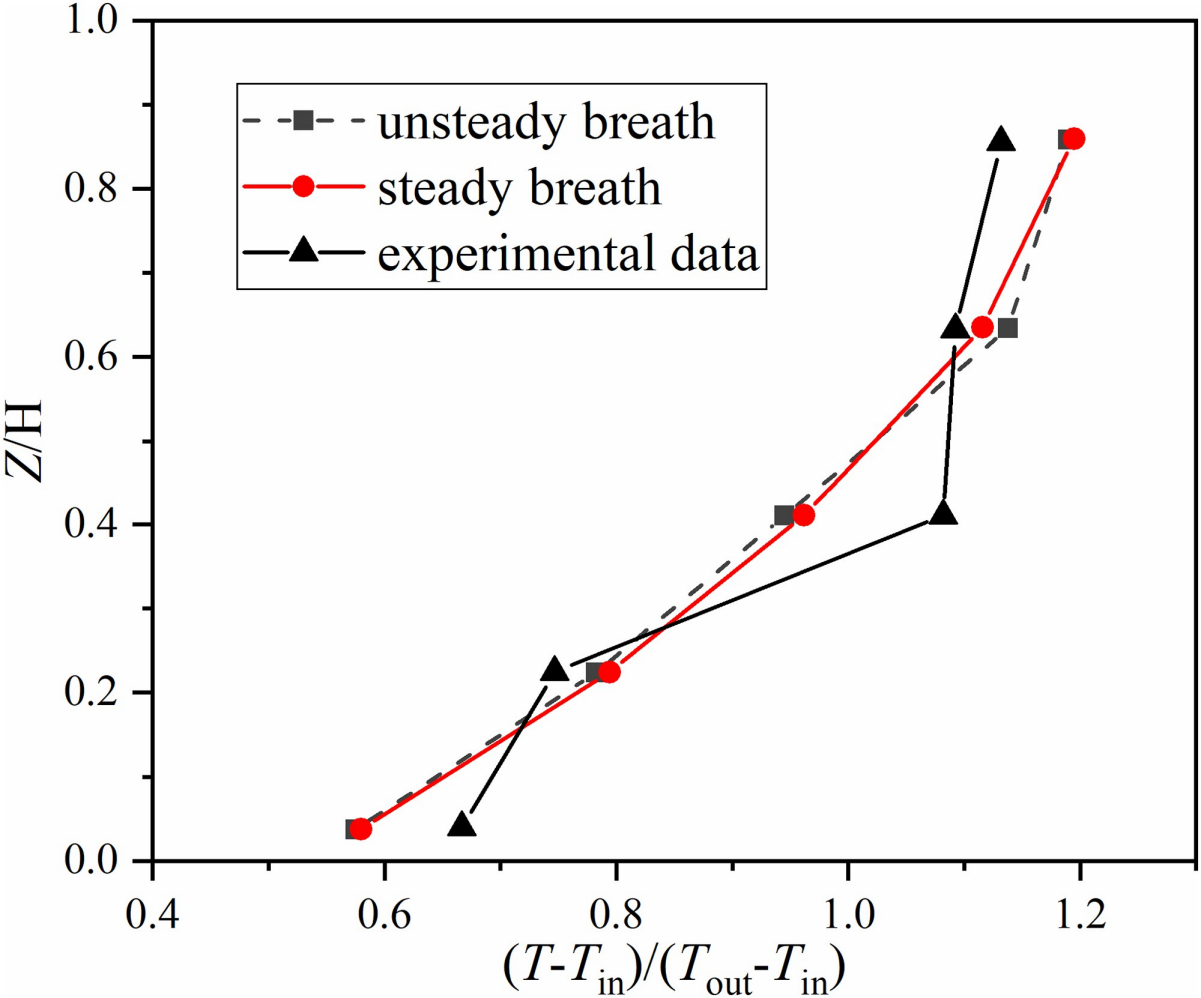
Simulated temperature gradients of the two boundary conditions and the experimental data. The reuse of experimental data has been authorized by Elsevier.

The comparison of the simulated temperature gradients among the two boundary conditions and the experimental data are shown in Fig 8. The sample points for temperature were at the coordinates of (3.7, 2.59, 0.1), (3.7, 2.59, 0.6), (3.7, 2.59, 1.1), (3.7, 2.59, 1.7), (3.7, 2.59, 2.3). In Fig 8, the horizontal axis represents (*T*-*T*_in_)/(*T*_out_-*T*_in_), where *T*_in_ is 16.1°C. The vertical axis shows the ratio of the sampling point’s height to the room’s height. The variation range of the simulated temperature gradient for both boundary conditions were larger than that of the measured temperature gradient.

**Fig 9:**
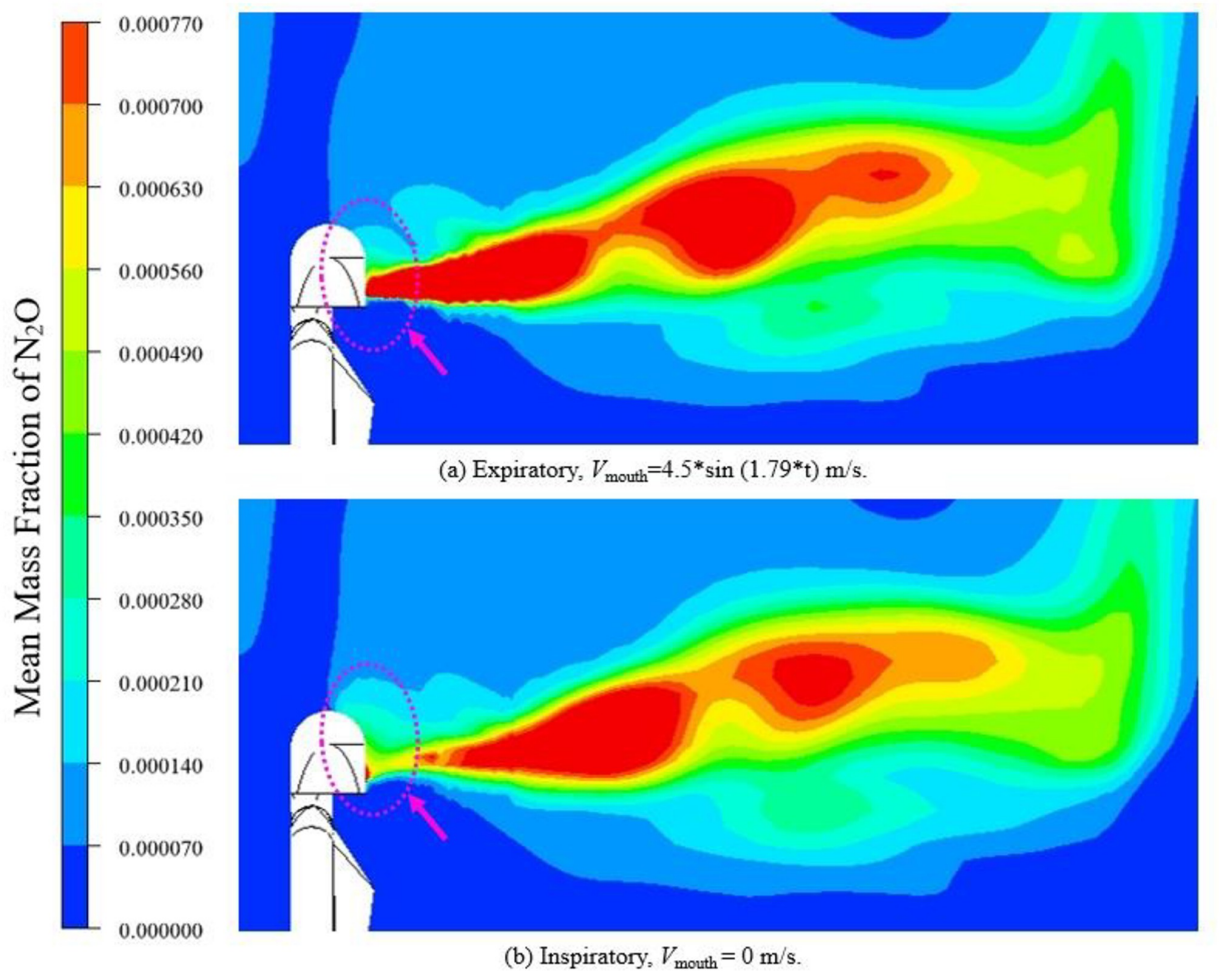
Mean N_2_O mass fraction during: (a) Expiratory phase and (b) Inspiratory phase.

In the experiment of this paper, N_2_O was used as a tracer gas. The transport of N_2_O concentration reflected the ability of exhaled gas to transport pollutants. With unsteady boundary conditions in both inspiratory phase and expiratory phase, the inspiratory was significantly more affected by thermal buoyancy. In Fig 9, the upper cloud image shows the mean N_2_O mass fraction during expiratory phase, while the lower cloud image shows mean N_2_O mass fraction during inspiratory phase. During the inspiratory phase, the concentration of the tracer gas near the mouth decreased, whereas the concentration near the upper part of the head increased. In contrast, the steady boundary conditions did not capture the changes in tracer gas concentration from the inspiratory phase to the expiratory phase.

**Fig 10:**
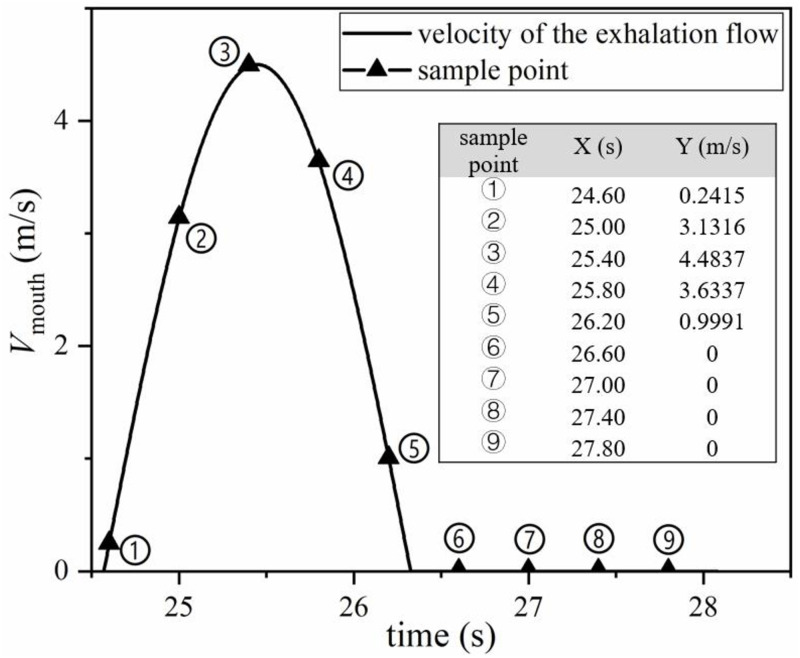
Sampling points during the 6th respiration.

To investigate changes in tracer gas concentration, particularly near the head, during complete respiration, nine sampling points were set up during the 6th complete breath, as shown in Fig 10. Among them, 5 sampling points were set up in the expiratory phase and 4 sampling points were set up in the inspiratory phase. The velocity of the exhalation flow at different times for sampling points are also shown in Fig 10. The maximum velocity point in the sampling point is ③, which is 4.4837 m/s at 25.4 s, the minim velocity is 0 m/s during the whole inspiration.

**Fig 11:**
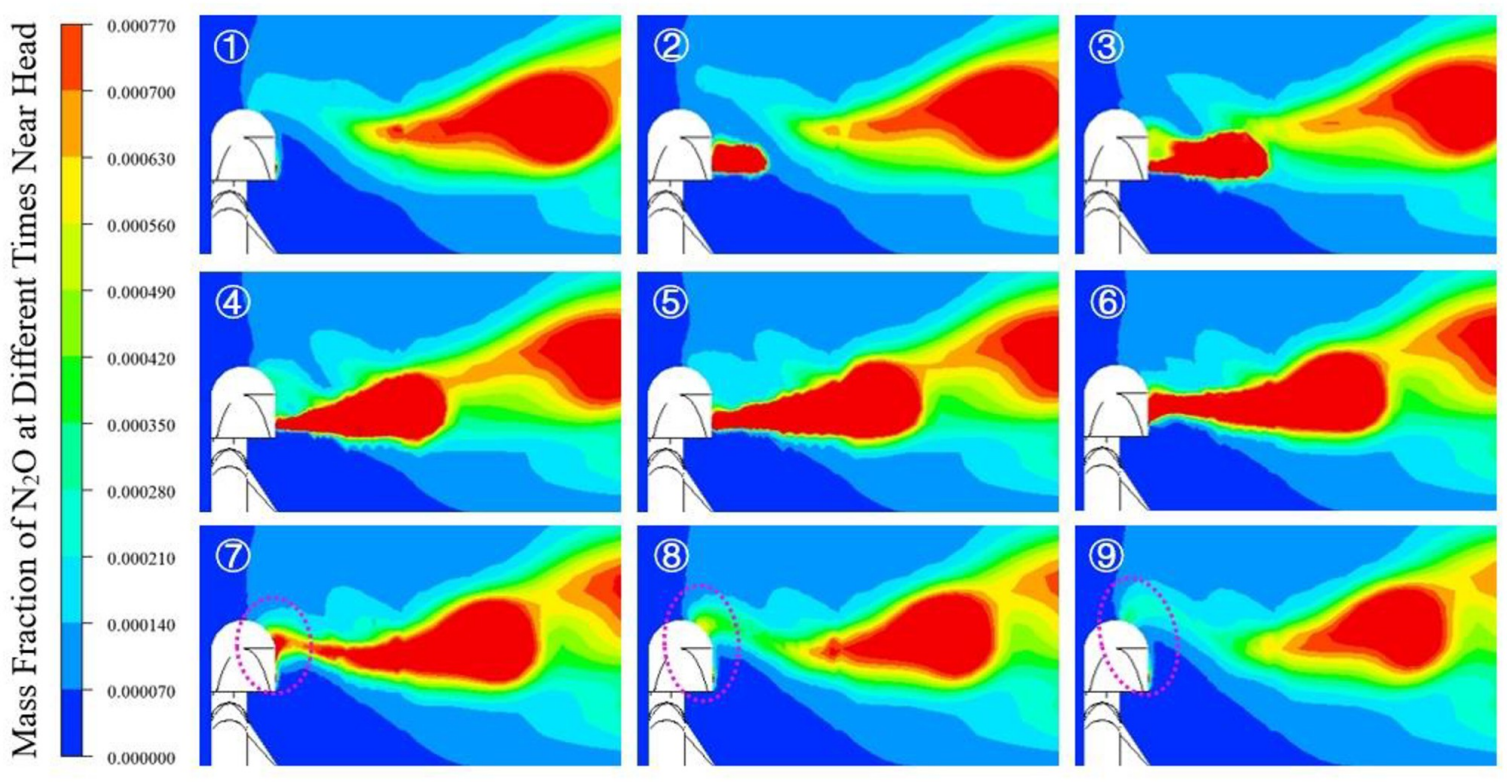
N_2_O mass fraction at 9 sampling points.

The cloud map of N_2_O mass fraction at 9 sampling points is shown in Fig 11. Under unstable conditions, N_2_O was transported in clumps along with exhaled gas. During the expiratory phase, a high concentration of tracer gas was exhaled from the mouth. During the inspiratory phase, the tracer gas near the mouth migrated to the upper head. At the end of the inspiratory phase, even at the beginning of the expiratory phase, the tracer gas raised further under the action of buoyancy, and it could reach to the top of the human head. The migration of pollutants during the inspiratory phase affects the human exposure risk, and it is necessary to use sinusoidal exhalation flow to simulate human respiration.

**Fig 12:**
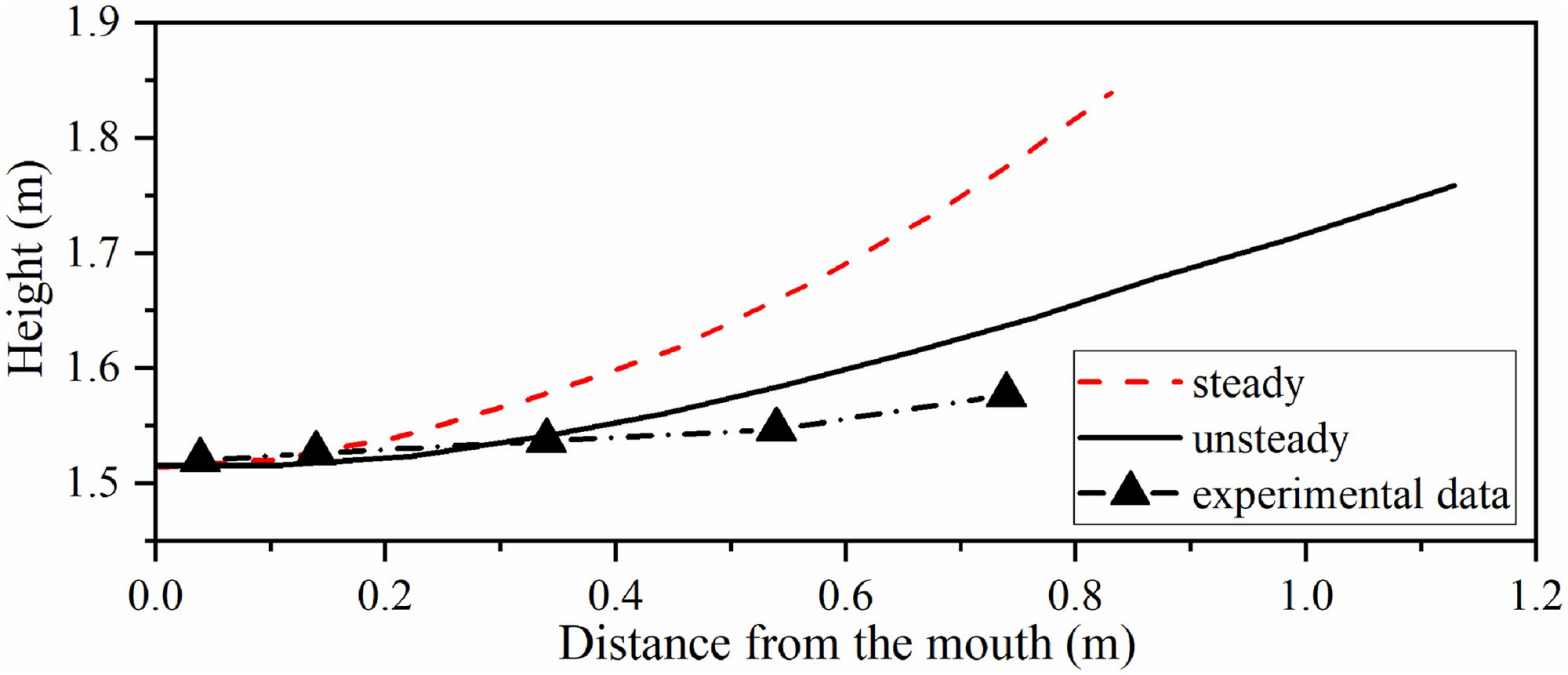
The track of jet centre under different boundary conditions. The reuse of experimental data has been authorized by Elsevier.

The axis of exhalation jet under steady boundary condition was different from that under unsteady boundary condition. Taking the highest velocity point of different x coordinates in jet as the track of the jet centre, and the result under different boundary conditions is shown in Fig 12. The unsteady sinusoidal velocity only flowed in expiratory phase and during inspiratory phase was 0 m/s. The steady boundary condition had the same integral flow volume in the whole respiration with constant velocity. Both the two boundary conditions were calculated for time-average velocity field in the 6th respiration. The track of jet centre could be obtained from the time-average field. The experimental data came from reference [23], and the results of unsteady simulation were close to Villafruela’ s simulation result.

### Discussion for different results between steady or unsteady boundary conditions

Results showed that in the region affected by the exhalation jet, there was a significant difference between the calculated results of steady/ unsteady boundary conditions, which can be explained by the underlying physics.

(1). The unsteady boundary condition can simulate the effect of buoyancy during inspiratory phase, in which the tracer gas in the mouth migrated to the upper part of the head. However, the steady case cannot reflect the buoyancy force of the pollutants during inspiratory phase.

The velocity of the exhaled air is the vector sum of the velocities in all directions, that is Eq (9):

$$\vec{V}=\vec{u}+\vec{v}+\vec{w}$$ (9)

The macroscopic velocity of the air mass is calculated as Eq (10):

$$\left|\vec{V}\right|=\sqrt{{\left(\left|\vec{u}\right|\right)}^{2}+{\left(\left|\vec{v}\right|\right)}^{2}+{\left(\left|\vec{w}\right|\right)}^{2}}$$ (10)

As in reference [34], the heat plume produced by the human face was about 0.2 m/s and was directed upwards. While the velocity of exhaled gas in steady state was 2.25 m/s forward. Therefore, under steady state near the mouth, the upward movement of the exhaled gas was not obvious. Under unsteady boundary conditions, during inspiratory phase, no gas was exhaled from the mouth, so the pollutants near the face were driven by the plume and move up the head. That’s the reason for the different transport results of pollutants.

(2). Under steady or unsteady boundary conditions, the penetration depth of exhalation jet was different. With the same expiratory flow volume, the maximum speed of exhaled gas was different in the two boundary conditions. Under unsteady case, the sinusoidal exhaled velocity was 4.5·sin (1.79·t) m/s, and the maximum velocity was 4.5 m/s. While under steady case, the constant velocity was 2.25 m/s, and so was the maximum velocity. The kinetic energy of the exhaled gas is Eq (11):

$$E_{k}=\frac{1}{2}mv^{2}$$ (11)

The maximum speed of sinusoidal exhalation has more kinetic energy. It can overcome more resistance in the flow, and that made it achieve more penetration depth. This may affect the accuracy of pollution exposure risk. Moreover, under the complex interaction among respiratory flow, thermal boundary laminar flow and environment thermal plume, the tracks of the jet centre by two boundary conditions were different.

Although using unsteady boundary conditions to calculate time-average field was much more computationally calculated than steady boundary conditions, it is necessary to use sinusoidal exhaled boundary condition for human respiration simulation, especially to study in the area affected by the exhalation jet.

### Switching flow character models in breathing simulation

If the airflow flows out of the source, it is likely to produce turbulence under the action of air viscosity, while if the gas flows into the sink under the action of pressure, in many cases, it would maintain laminar flow. Human breathing contains both source and sink. This section verifies the simulation strategy of switching flow model according to the respiratory boundary conditions in literature [23]. During the sinusoidal expiratory phase, the RNG *k-ε* model including a differential formula for effective viscosity to account for low-Reynolds-number was selected. For the inspiratory phase, where the exhaled gas velocity was 0 m/s, both the previous RNG *k-ε* model and the laminar flow were respectively calculated for comparison. Each of the two simulation strategies calculated 6 breathing cycles. In fact, by the end of the 5th breathing cycle, the effect of the initial value could already be eliminated.

Since the coupled algorithm takes a huge amount of computation to calculate unsteady simulation, and most of current literatures use the separation algorithm for unsteady simulation, the SIMPLE algorithm was used in this section to verify the simulation strategy of switching flow model. The SIMPLE algorithm is widely used in both commercial and open-source CFD software and is known for its reliability and accuracy in unsteady simulations. The SIMPLE algorithm is an iterative algorithm, and non-iterative algorithms can also be used for unsteady simulations to reduce the computation time. The selection of algorithms will be discussed in the next section.

The results of switching flow model and the only RNG *k-ε* model are shown in Table 3. The values in Table 3 were reserved for 4 significant digits after the decimal point. The outlet flow of the two calculation results was the same, the mean N_2_O mass fraction had a tiny deviation, the mean indoor temperature and mean static pressure had a small deviation. In order to accurately obtain the effects of different simulation strategies on vorticity and velocity, the vorticity magnitude and velocity magnitude were calculated on the y = 1.595 m plane, which was the symmetric plane of the Y axis of the room. The maximum value of vorticity and velocity in the room were both in this plane, and each physical parameter had a considerable range of variation. The real time of the result was at t = 21.06 s, and that was the end time of the 6th respiratory cycle. Results showed that the average vorticity deviation was 41% between switching flow model and the only RNG *k-ε* model, while the average velocity deviation was much smaller, only 2%. That’s because the turbulent dissipation was considered in RNG *k-ε* model, and the vorticity of exhaled gas was dissipated during the inspiratory phase, which also led to a decrease in the average velocity of exhaled gas. Under the sinusoidal exhaled boundary condition, the exhaled gas velocity had experienced a process from peak to 0 before inspiration, and simulating in laminar flow can better preserve the vorticity of exhaled gas during inspiration. In the built environment without other disturbances, the vorticity can be preserved for a certain period.

**Table 3:** Comparison results of switching flow model and only RNG *k-ε* model.

	Time-average of the 6th complete respiratory cycle				End time of the 6th respiratory cycle, t = 21.06s, symmetric plane, y = 1.595m	
	Mean indoor temperature [°C]	Mean mass fraction of N_2_O	mean static pressure [Pa]	outlet flow [kg/s]	vorticity magnitude [s ^[–1]^]	velocity magnitude [m/s]
switching flow model	20.1988	0.00005665	0.7087	-0.06646	1.3372	0.07739
only RNG *k-ε* model	20.1988	0.00005664	0.7095	-0.06646	0.7838	0.07590

**Fig 13:**
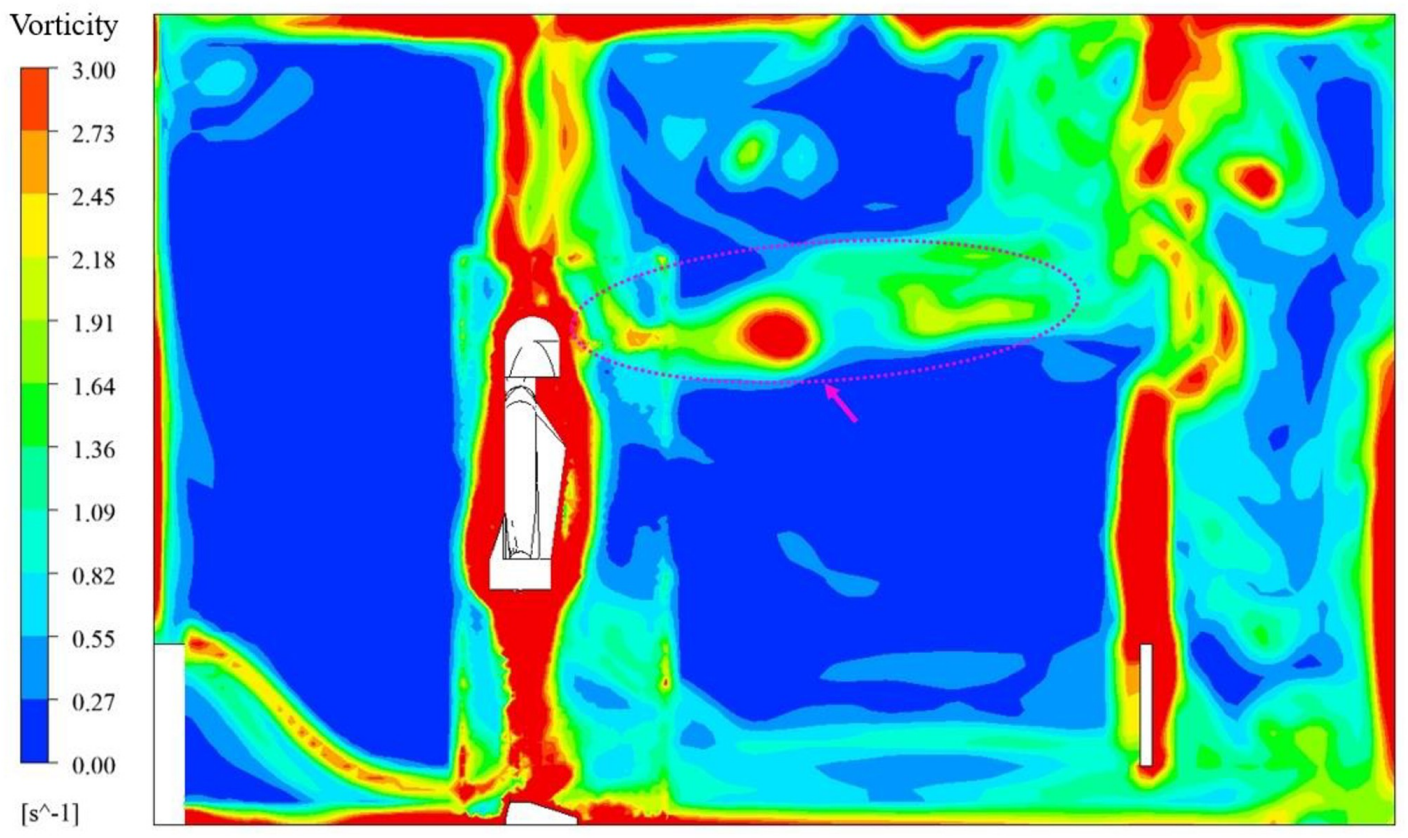
Vorticity for RNG *k-ε*+laminar model, t = 21.06 s.

**Fig 14:**
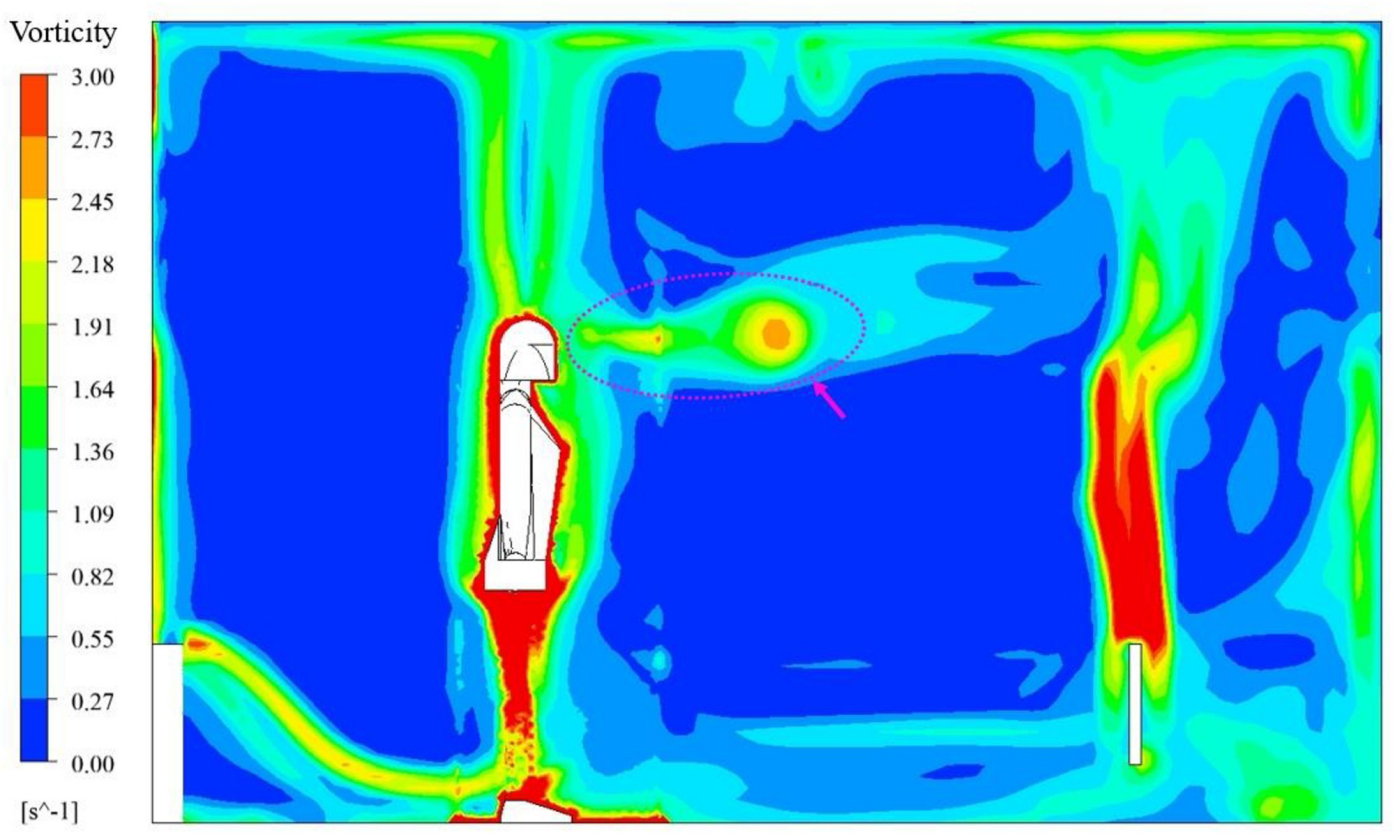
Vorticity for only RNG *k-ε* model, t = 21.06 s.

For the end time of the 6th respiratory cycle, t = 21.06s, on the symmetric plane, y = 1.595 m, the cloud image of vorticity for RNG *k-ε*+laminar model is shown in Fig 13, the cloud image of vorticity for only RNG *k-ε* model is shown in Fig 14. As can be seen from the two figures, with RNG *k-ε*+laminar model, the vorticity of exhaled gas was enhanced, and the penetration depths of exhaled gas was also increased. Since the laminar model does not take turbulent dissipation into account, the exhalation jet can maintain more kinetic energy and penetrate deeper. The change of penetration depths can affect the assessment of human exposure risk, and the increased depth can increase the risk of exposure.

**Fig 15:**
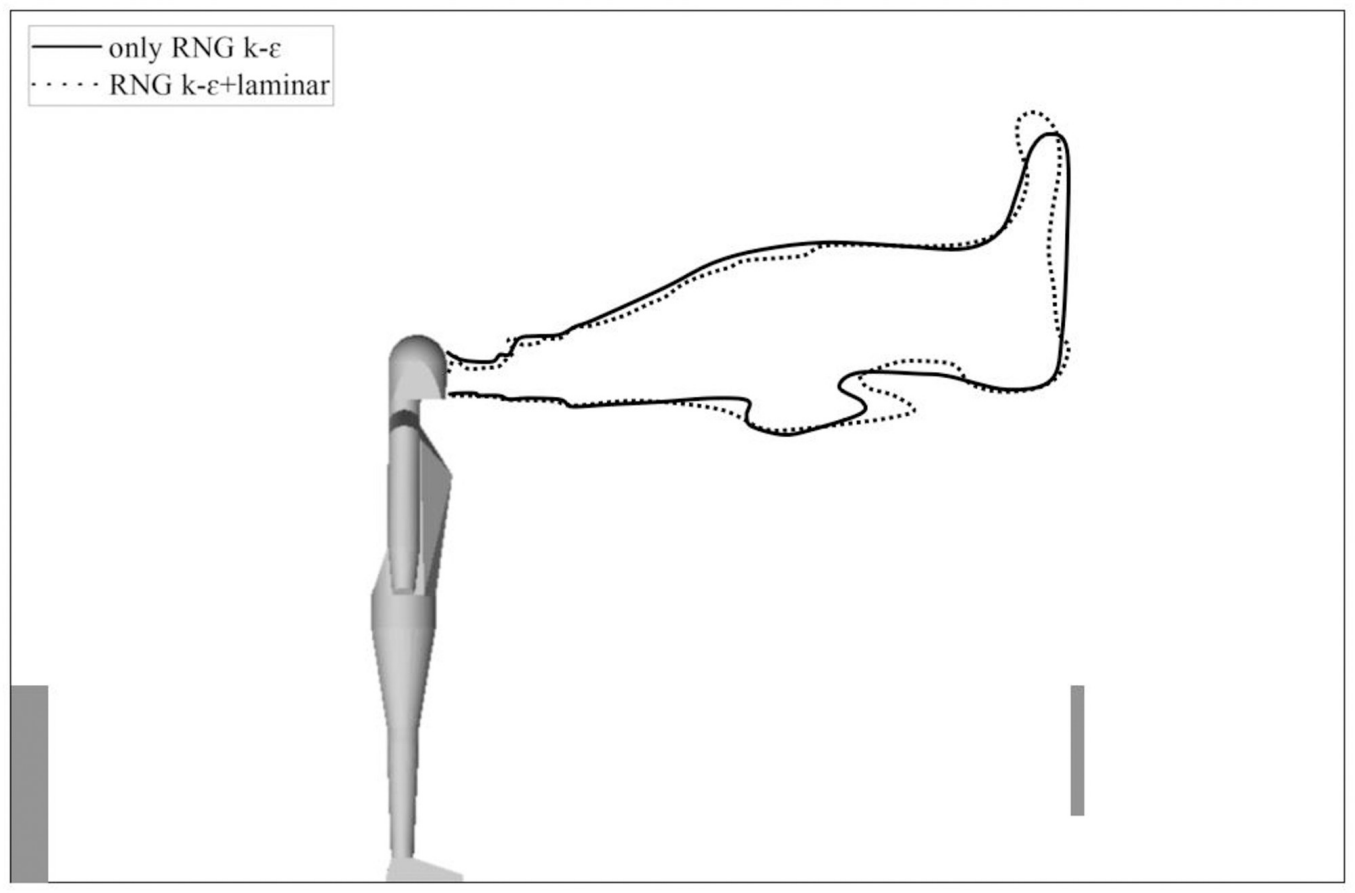
Cloud image of 1% mass fraction N_2_O under different flow model strategies.

In the time-average flow field of the 6th complete respiratory cycle, the cloud image of 1% mass fraction N_2_O under different flow model strategies is shown in Fig 15. In the unsteady simulation, the simulation period of real time was 17.56–21.06 s. The N_2_O concentration of 1% mass fraction was already very small, and the cloud images obtained by the two simulation strategies were roughly similar. With only RNG *k-ε* model, the effect of buoyancy at the upper part of the head was more obvious, while using laminar model during inspiration could better maintain vorticity in exhaled gas. The difference between the two strategies were within an acceptable range for engineering accuracy.

**Fig 16:**
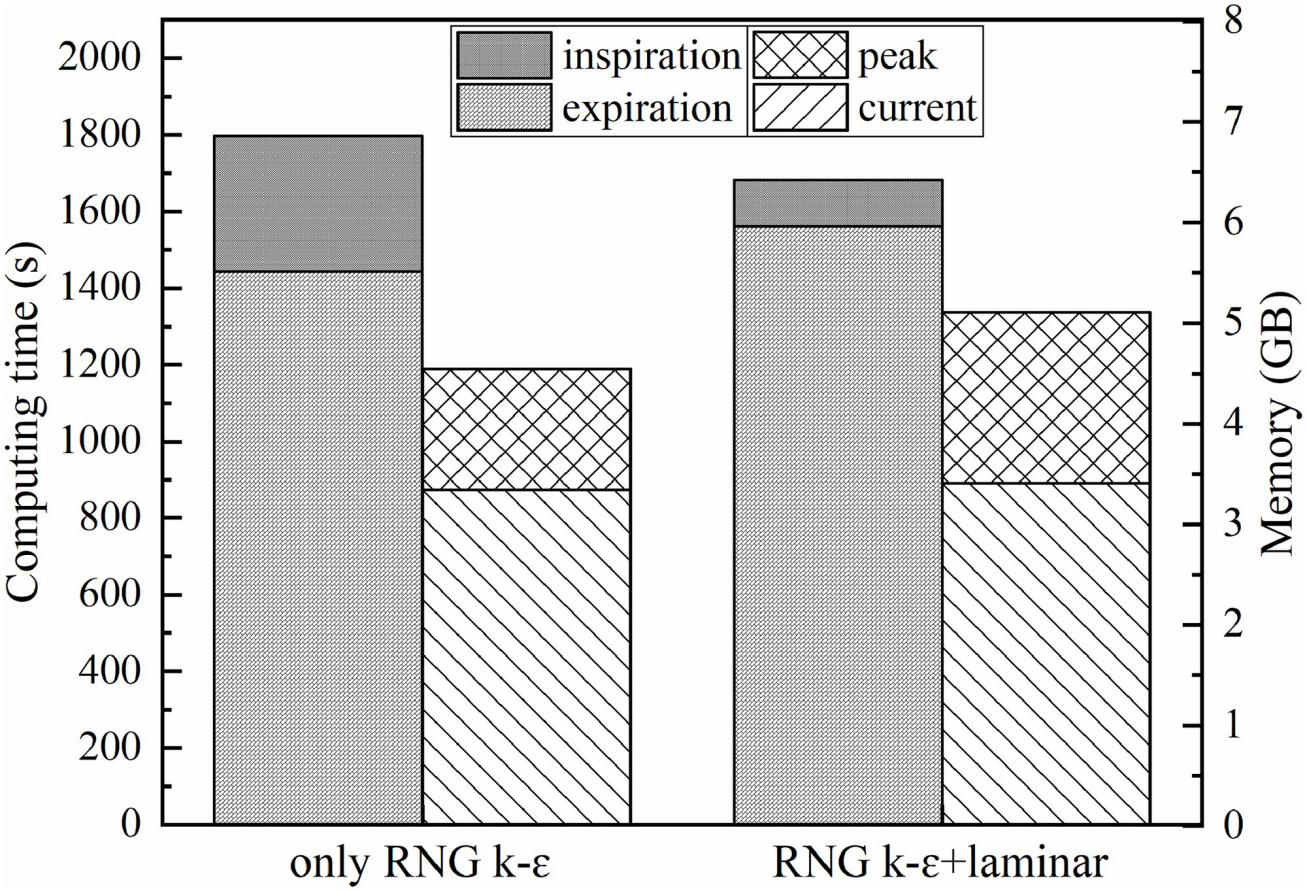
Computation time and memory usage of two simulation strategies.

The calculation time and occupied memory of the two simulation strategies are shown in Fig 16. Two groups, the only RNG *k-ε* model and the RNG *k-ε*+laminar model, simulated the 6th complete respiration cycle, respectively. The left axis is calculation time, which measures the calculation time used by computer to simulate expiration and inspiration; The right axis is memory usage, including current memory at the end of the simulation and ever gained peak memory during the full calculation. As can be seen from the Fig 16, in the inspiratory phase, the calculation time of both methods was much smaller than that of the expiratory phase, that’s because a lot of calculation was reduced when the velocity of exhaled gas from mouth becomes 0 m/s. If the laminar model was used instead of the RNG *k-ε* model to simulate the inspiratory phase, the calculation time for inspiratory phase could be reduced by about 66%. After switching the flow character model to laminar, the calculation time for expiratory phase increased by 8% than only using the RNG *k-ε* model, but the calculation time for the entire respiration decreased by 6%. With this switching strategy, the time saved in the inspiratory phase was greater than the time gained in the expiratory phase.

In Fig 16, the sum of peak and current indicates the maximum memory ever occupied in entire breathing cycle. As shown in Fig 16, the maximum memory occupied of RNG *k-ε*+laminar simulation strategy was about 12% higher than that of only RNG *k-ε* model.

The computer model used in this article was the Dell OptiPlex Tower Plus 7010, which contains an i7-13700 2.1GHz CPU and 16GB of 4400MHz RAM.

### Non-iterative algorithm combined with switching flow model simulation strategy

**Fig 17:**
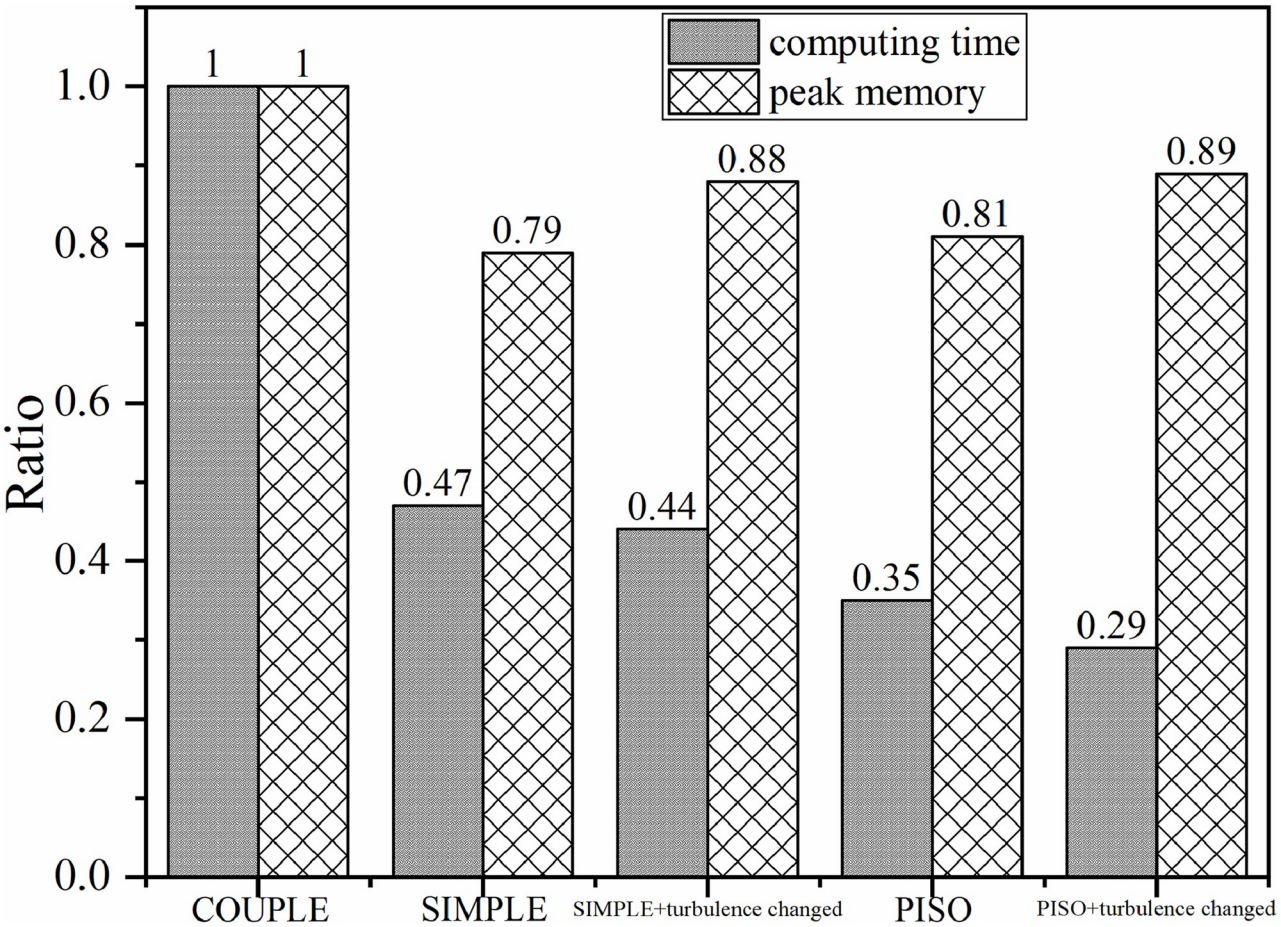
Comparison of calculation time and memory occupied of different simulation strategies.

In this paper, for the 6th complete human respiration cycle, the comparison of calculation time and occupied memory for simulation using different algorithms combined with only RNG *k-ε* model and RNG *k-ε*+laminar model are shown in Fig 17.

In Fig 17, peak memory means the maximum memory ever required during the 6th respiratory cycle simulation. Using the coupled algorithm with only RNG *k-ε* model, the calculation time and the peak memory required for the 6th respiration cycle simulation were taken as reference values, and the ratio of computing resources required for different simulation strategies was calculated. The ordinate of the figure is the scale, ranging from 0–1. As Fig 17 shows, the calculation time and peak memory required by the separate algorithm in unsteady simulation were smaller than that of the coupled algorithm. Under the same simulation strategy, the computational time required by iterative algorithm was more than that of non-iterative algorithm, and the peak memory required by iterative algorithm was almost the same but a little lower than that of non-iterative algorithm. No matter use iteration algorithm or non-iteration algorithm, if the simulation strategy of switching flow model was adopted, the calculation time would be decreased, but the peak memory required for calculation would be increased. If the PISO algorithm was used for calculation with the simulation strategy of switching flow models, the calculation time would be significantly reduced.

**Fig 18:**
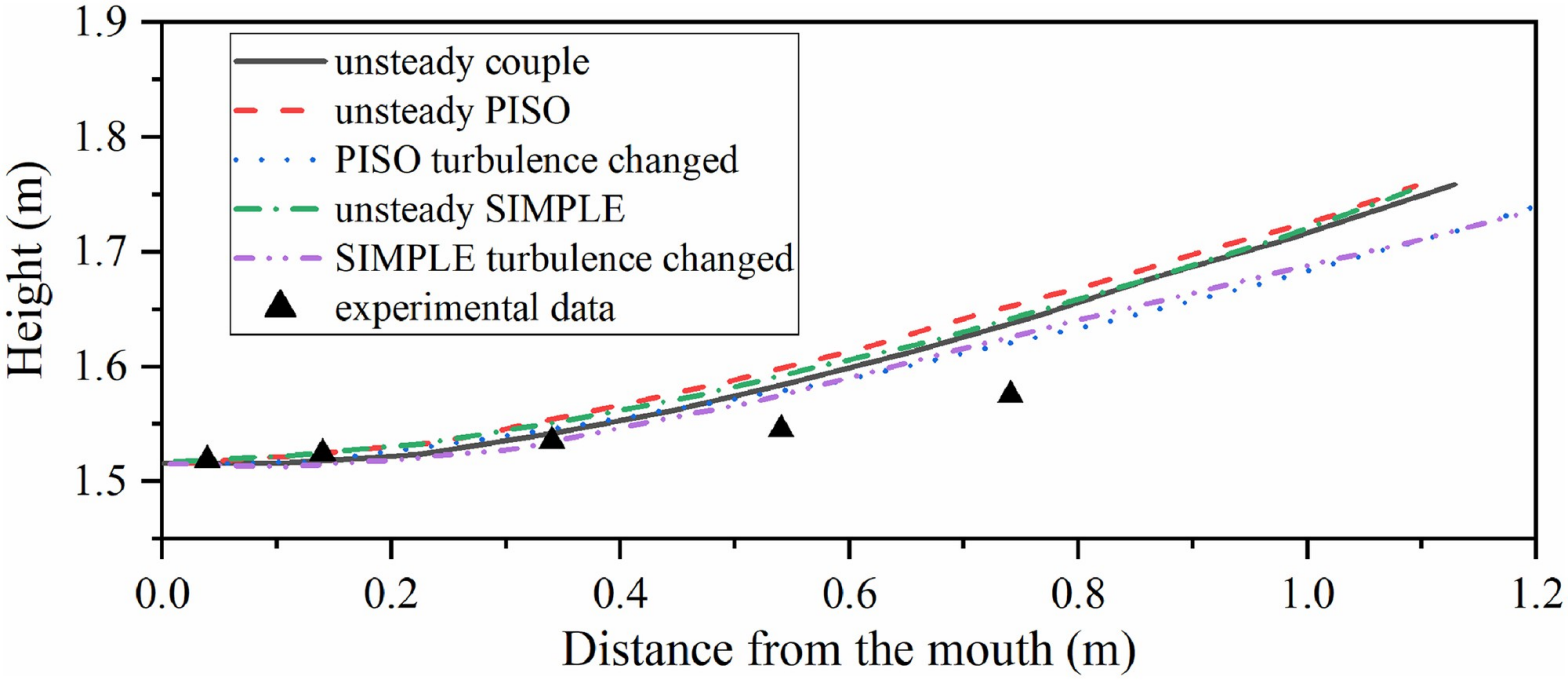
Jet centre track under different simulation strategies. The reuse of experimental data has been authorized by Elsevier.

Taking the highest velocity point of different x coordinates in jet as the track of the jet centre, in the time-average of 6th complete human respiratory cycle, the jet centre track under different simulation strategies is shown in Fig 18.

In Fig 18, both the SIMPLE algorithm and PISO algorithm show that the jet track was closer to the measured value after using the simulation strategy of switching flow model. That was because the turbulent dissipation was not considered in laminar model for the exhaled gas during inspiratory phase, so the penetration depth of the jet was longer and the influence of thermal buoyancy was weakened. After using the simulation strategy of switching flow model, the calculation results of PISO algorithm were similar to those of SIMPLE algorithm, which proved the reliability of the calculation results. In fact, if the laminar model was used to simulate the inspiratory phase, the calculation process would be more convergent than the only RNG *k-ε* model.

Therefore, it is considered in this section that PISO algorithm with RNG *k-ε*+laminar model can be used to simulate human respiration. This simulation strategy is a good choice with reliable calculation results and less calculation time.

## Conclusions

Given the significance of human respiration in the study of indoor environments and human health, this paper uses the experiment described in literature [23] as a case study to propose a CFD-based simulation strategy for human respiration. The proposed simulation strategy is as follows:

1. Using unsteady boundary conditions to simulate human respiration. Unlike previous studies, the coupled algorithm was used in this paper and finally got the convergence solution under steady boundary conditions with thermal buoyancy. However, the simulation results of different boundary conditions were significantly different:
(1). Since the exhaled gas velocity under constant expiratory flow was less than the maximum velocity in sinusoidal exhaled velocity, the maximum kinetic energy of exhaled gas is different between the two boundary conditions, and so is the jet penetration depth. Under the complex interaction among respiratory flow, thermal boundary laminar flow and environment thermal plume, the exhalation jet centre of the two boundary conditions were different.
(2). Unsteady boundary conditions were able to simulate buoyancy during the inspiratory phase, in which the tracer gas around the mouth migrated to the upper part of the head. The steady-state boundary conditions could not reflect the migration of pollutants near the mouth during inspiratory phase. Detailed physical explanations are in the previous section.
2. Using the RNG *k-ε*+laminar model to simulate human respiration, in which the RNG *k-ε* model was used during expiratory phase and the laminar model was used during inspiratory phase. The use of laminar model could better preserve the vorticity of exhaled gas and increase the penetration depth of exhalation jet, which was more in line with the real physical phenomena. Compared to the RNG *k-ε* model, the laminar model could significantly reduced computation time and required fewer iterations. Although switching laminar model would increase the occupied memory, the strategy of switching flow character model is still friendly and feasible at the present in which the computer performance surplus. In the simulation results of N_2_O transportation, the cloud images obtained by the two methods were roughly similar, and both of them could meet the requirements of engineering accuracy.

In terms of calculation accuracy, the laminar flow model does not account for turbulent dissipation, so the kinetic energy of the exhaled gas can be better maintained, and the penetration depth of the exhalation jet can be increased. This change in penetration depths can affect the assessment of human exposure risk, as the increased depth can increase the risk of exposure.

3. Using PISO algorithm combined with RNG *k-ε*+laminar model. PISO algorithm is a non-iterative algorithm, which can reduce the calculation time and obtain reliable results when applied to unsteady simulation. The stability of PISO algorithm is stronger than that of SIMPLE algorithm, and the memory occupied is almost the same but a little larger than that of SIMPLE algorithm. The strategy of using PISO algorithm combined with RNG *k-ε*+laminar model in human respiration simulation is the optimal one in this paper, and it can conduct to reliable calculation results and less calculation time.

For rural houses in Inner Mongolia, China, the optimal strategy selected in this paper can be used to simulate the indoor risk of exposure to respiratory pollutants. The case in this paper considers thermal plume of radiator and human body, and it is suitable for indoor environment simulation in winter. Similarly, this paper is also applicable to the assessment of the exposure risk of exhaled pollutants in the Winter Olympic stadium.

Research deficiency and prospect: The optimization of the human respiration simulation still needs improvement, and the goal of improvement is still to improve the calculation accuracy and reduce the calculation time. It is hoped that the projection algorithm proposed by Chorin [42] and Témam [43, 44], FFD [45] based on semi-Lagrange method [46], and AI-enhanced CFD, can be combined with RNG *k-ε*+laminar model in future studies to find better human respiration simulation strategies.

## Supporting information

S1 File Different strategy in Fig 18. (ZIP)

S1 Fig Steady simulation for Fig 12. (JPG)

S2 Fig Unsteady simulation for Fig 12. (JPG)

S3 Fig N2O mass fraction in Fig 15-only RNG. (JPG)

S4 Fig N2O mass fraction in Fig 15-turbulence changed. (JPG)

S1 Table Data in Fig 7. (XLSX)

S2 Table Data in Fig 8. (XLSX)

S3 Table Data in Fig 10. (XLSX)

S4 Table Data in Fig 16. (XLSX)

S5 Table Data logging in Fig 16. (XLSX)

S6 Table Data in Fig 17. (XLSX)

S7 Table Data logging in Fig 17. (XLSX)

## Abbreviations

$\left|\vec{u}\right|$: The magnitude of the velocity vector in the x direction
$\vec{V}$: Velocity vector in space
$\vec{u}$: Component of the velocity vector in the x direction
∇: The Hamiltonian operator
∇^2^: The Hessian Matrix
A: The diagonal matrix obtained by matrix M decomposition
CSPs: Computer simulated persons
E_k_: Kinetic energy, unit: J
f: Source term in the N-S equation
M: The discretization coefficient matrix for momentum equation
P: Pressure, unit: Pa
PMV: Predicted Mean Vote, used to evaluate human thermal comfort
PPD: Predicted percentage of dissatisfied, used to evaluate human thermal comfort
T: Air temperature, unit: °C
t: Time, unit: s
T_in_: Temperature of gas supplied by the diffuser, unit: °C
T_out_: Temperature of gas discharged through the exhaust, unit: °C
U: Average velocity in the X direction, unit: m/s
*u*’: Pulsation velocity in the X direction, unit: m/s
u: The speed in the X direction, scalar quantity, unit: m/s
V: Average velocity in the Y direction, unit: m/s
v: Kinematic viscosity, unit: m^2^/s
*v*’: Pulsation velocity in the Y direction, unit: m/s
v: The speed in the Y direction, scalar quantity, unit: m/s
V_air_: The speed of air flow at sampling point
V_mouth_: The speed of exhaled gas at the mouth
W: Average velocity in the Z direction, unit: m/s
*w*’: Pulsation velocity in the Z direction, unit: m/s
w: The speed in the Z direction, scalar quantity, unit: m/s
ρ: Density, unit: kg/m³
