# Supplementary figures and images for: Optimal human respiratory simulation for exhaled gas based on CFD method
Source: PLoS One. 2024 Nov 18;19(11):e0313522. doi: 10.1371/journal.pone.0313522 (PMC11573226; doi:10.1371/journal.pone.0313522)

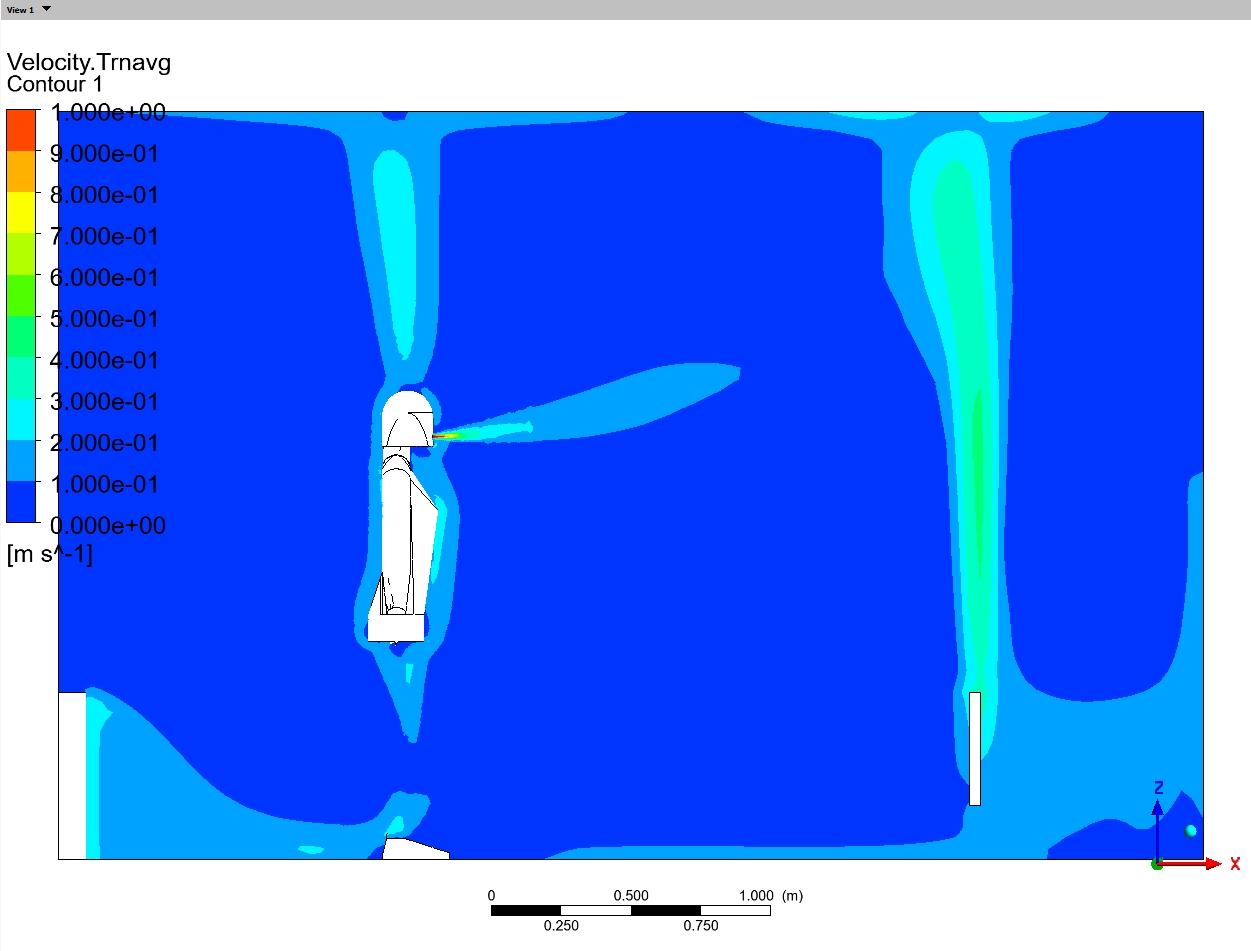

Supplement: S1 File — (ZIP) [file pone.0313522.s001.zip › Couple Transient.png]

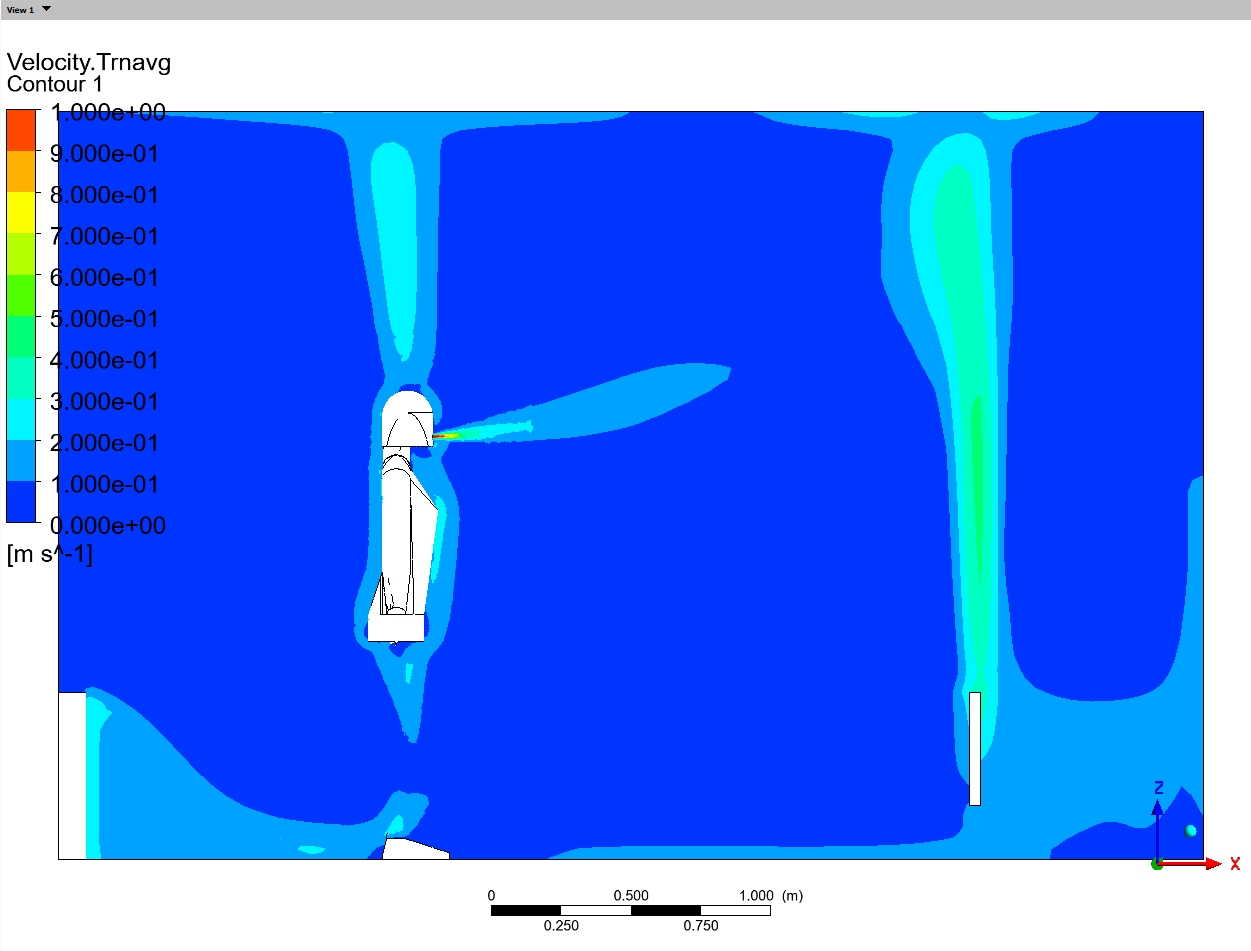

Supplement: S1 File — (ZIP) [file pone.0313522.s001.zip › Piso Transient.png]

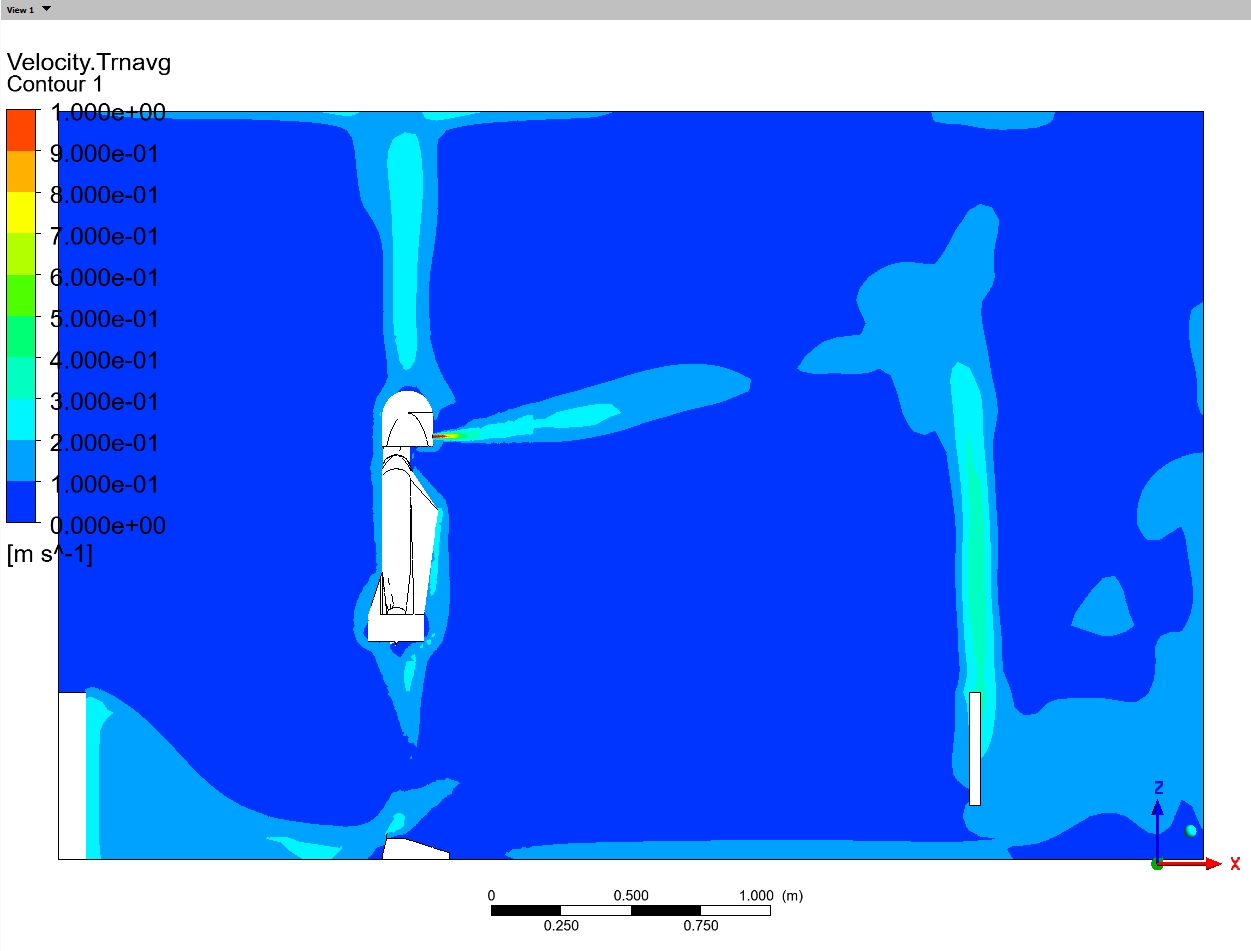

Supplement: S1 File — (ZIP) [file pone.0313522.s001.zip › Piso Turbulence Changed.png]

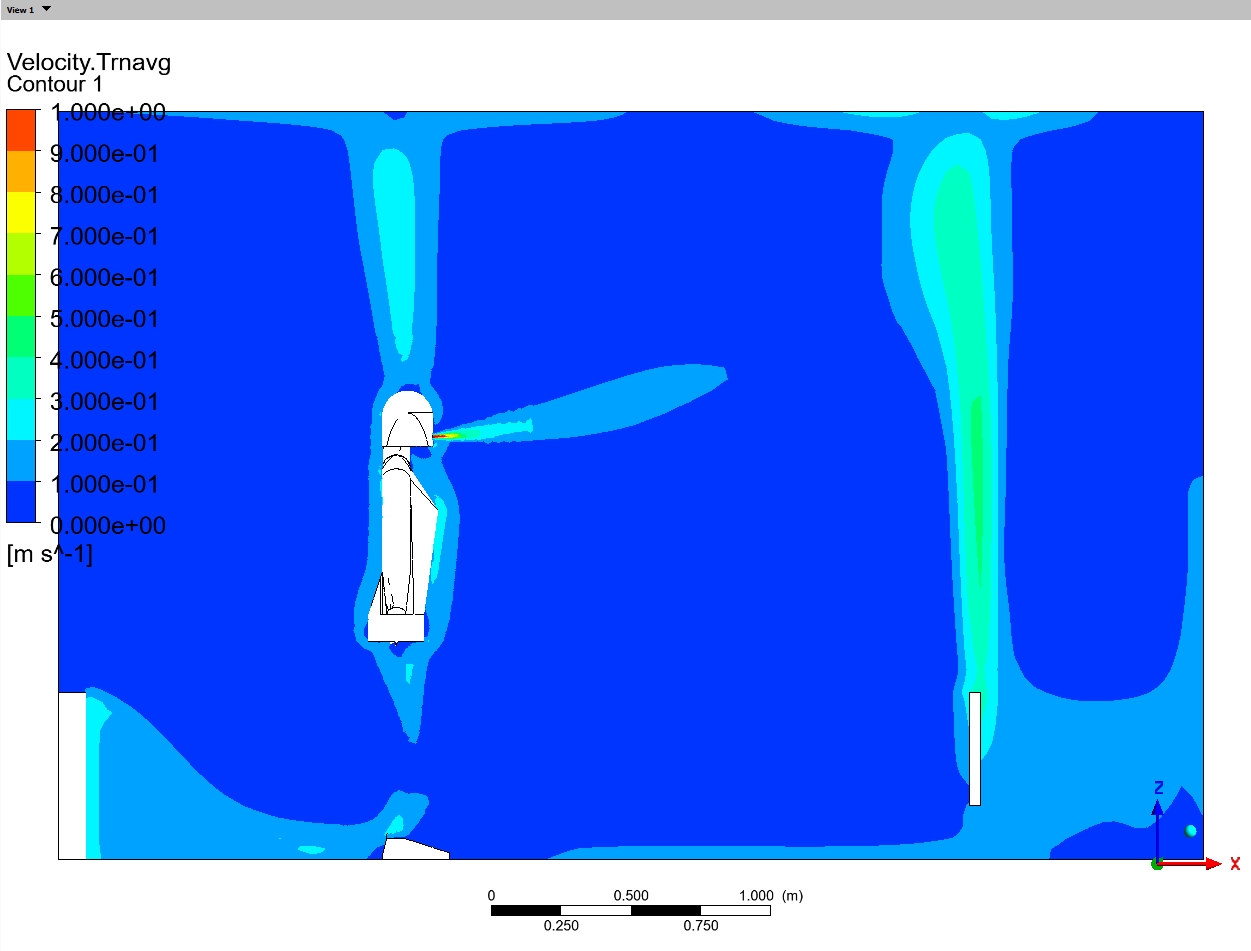

Supplement: S1 File — (ZIP) [file pone.0313522.s001.zip › Simple Transient.png]

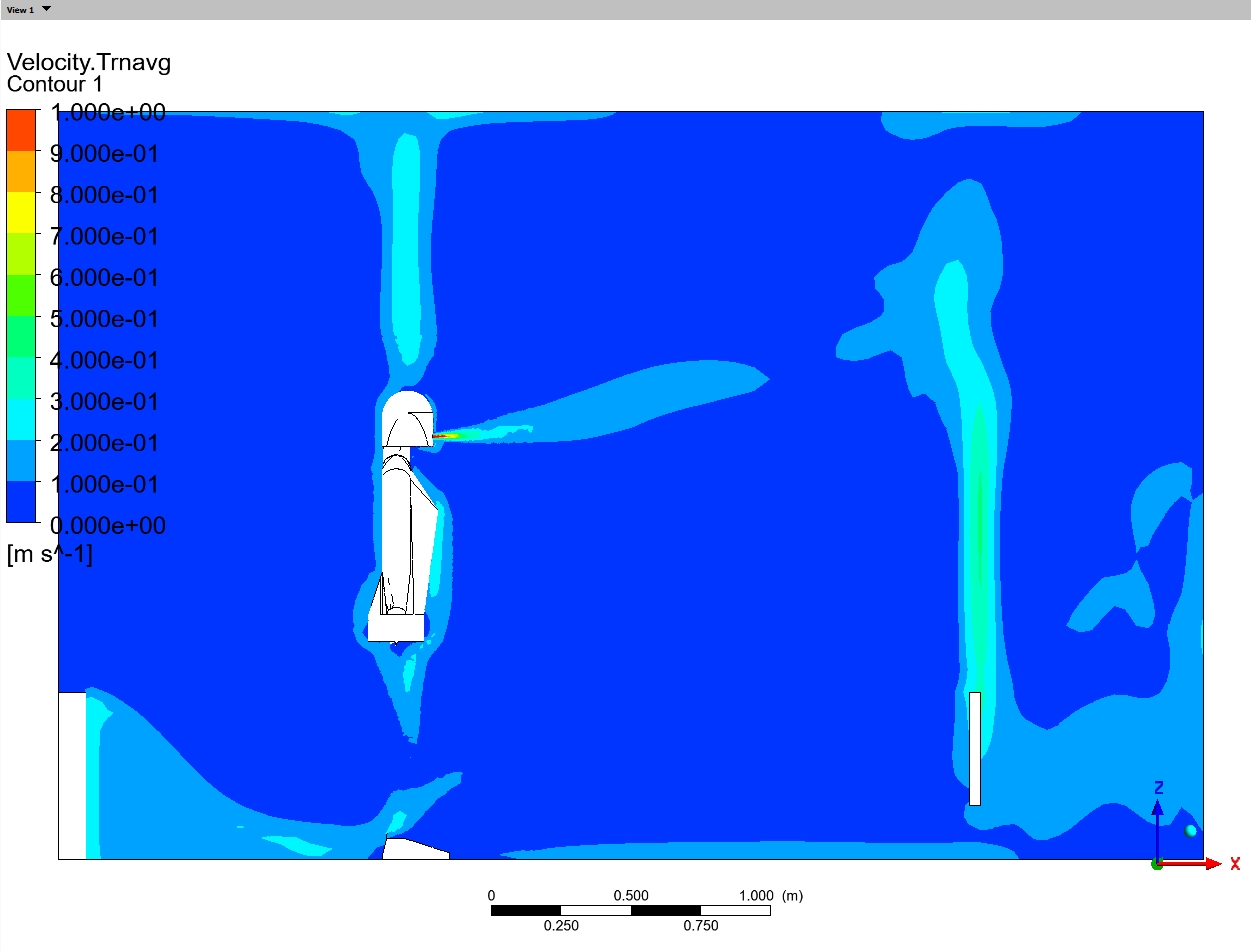

Supplement: S1 File — (ZIP) [file pone.0313522.s001.zip › Simple Turbulence Changed.png]

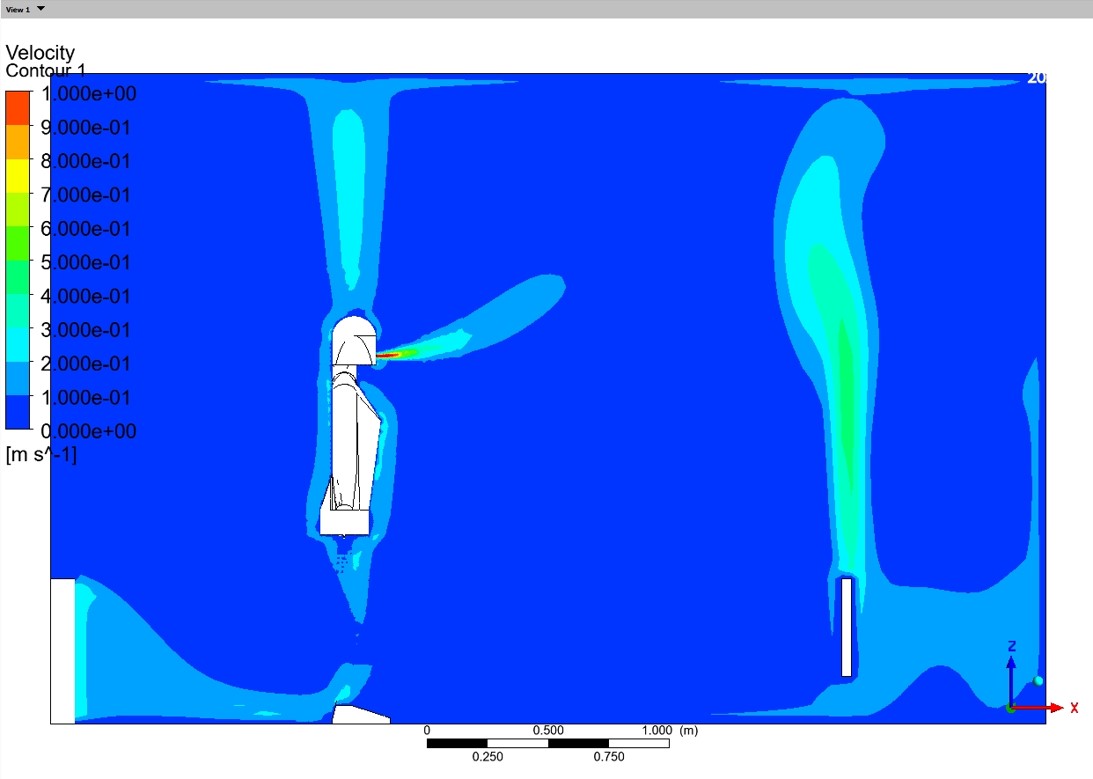

Supplement: S1 Fig — (JPG) [file pone.0313522.s002.jpg]

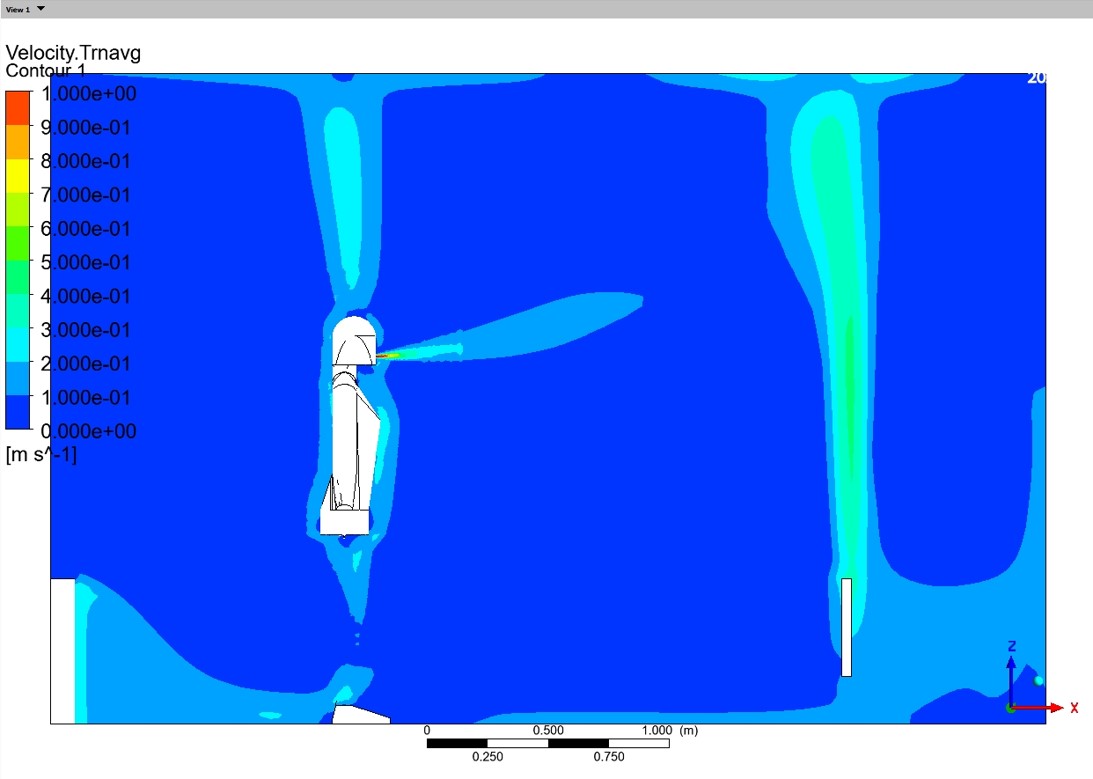

Supplement: S2 Fig — (JPG) [file pone.0313522.s003.jpg]

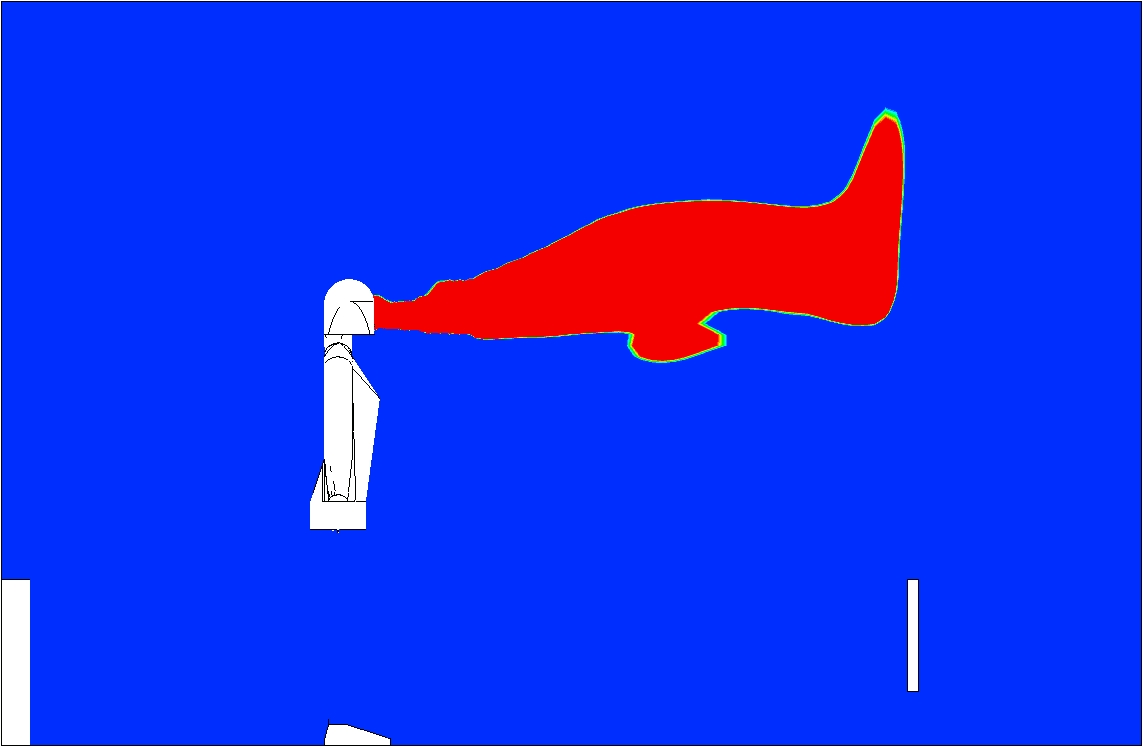

Supplement: S3 Fig — (JPG) [file pone.0313522.s004.jpg]

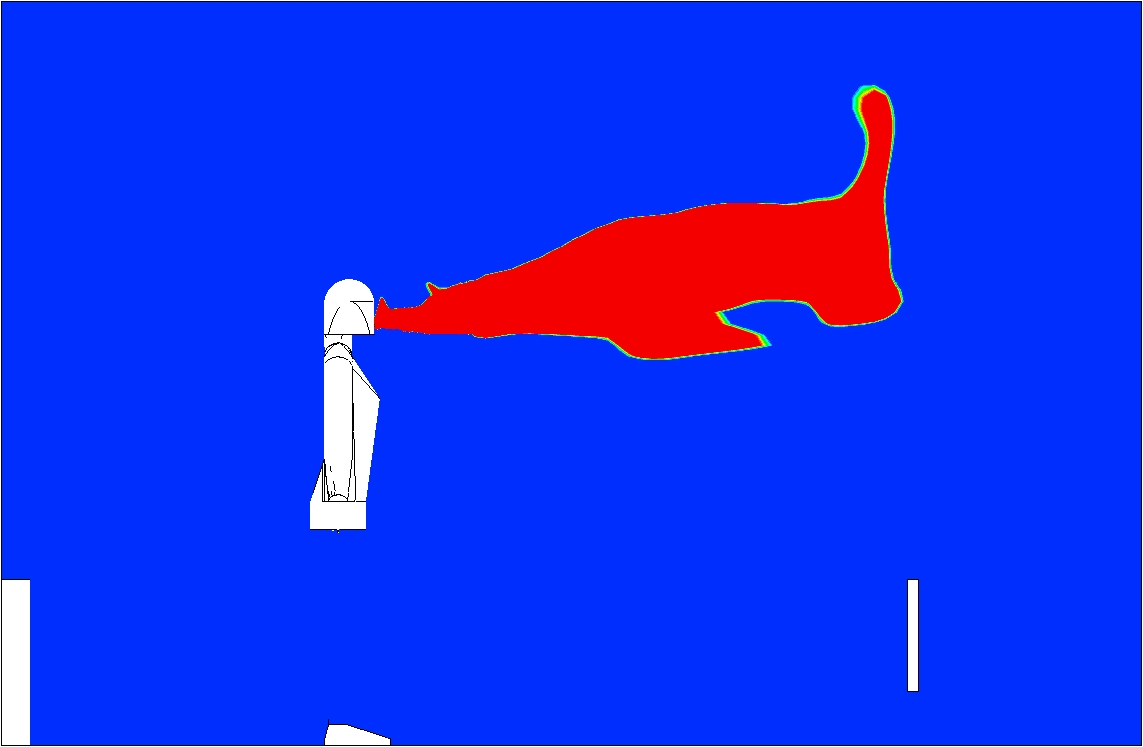

Supplement: S4 Fig — (JPG) [file pone.0313522.s005.jpg]
